# Immune-related gene characterization and biological mechanisms in major depressive disorder revealed based on transcriptomics and network pharmacology

**DOI:** 10.3389/fpsyt.2024.1485957

**Published:** 2024-12-06

**Authors:** Shasha Wu, Qing Jiang, Jinhui Wang, Daming Wu, Yan Ren

**Affiliations:** ^1^ Department of Psychiatry, Shanxi Bethune Hospital, Shanxi Academy of Medical Sciences, Tongji Shanxi Hospital, Third Hospital of Shanxi Medical University, Taiyuan, China; ^2^ Tongji Hospital, Tongji Medical College, Huazhong University of Science and Technology, Wuhan, China; ^3^ Third Hospital of Shanxi Medical University, Shanxi Bethune Hospital, Shanxi Academy of Medical Sciences, Tongji Shanxi Hospital, Taiyuan, China; ^4^ Department of Pharmacy, Shanxi Medical University, Taiyuan, China; ^5^ Academy of Medical Sciences, Shanxi Medical University, Taiyuan, China; ^6^ Department of Psychiatry, Xiaoyi City Central Hospital, Xiaoyi, China; ^7^ Department of Psychiatry, The Fifth Hospital of Shanxi Medical University, The Fifth Clinical Medical College of Shanxi Medical University, Shanxi Provincial People’s Hospital, Taiyuan, China

**Keywords:** major depressive disorder, immune-related hub genes, diagnostic, network pharmacology, bioinformatics

## Abstract

**Background:**

Major depressive disorder (MDD) is a severe psychiatric disorder characterized by complex etiology, with genetic determinants that are not fully understood. The objective of this study was to investigate the pathogenesis of MDD and to explore its association with the immune system by identifying hub biomarkers using bioinformatics analyses and examining immune infiltrates in human autopsy samples.

**Methods:**

Gene microarray data were obtained from the Gene Expression Omnibus (GEO) datasets GSE32280, GSE76826, GSE98793, and GSE39653. Our approach included differential expression analysis, weighted gene co-expression network analysis (WGCNA), and protein-protein interaction (PPI) network analysis to identify hub genes associated with MDD. Subsequently, gene ontology (GO), Kyoto Encyclopedia of Genes and Genomes (KEGG), Cytoscape plugin CluGO, and Gene Set Enrichment Analysis (GSEA) were utilized to identify immune-related genes. The final selection of immune-related hub genes was determined through the least absolute shrinkage and selection operator (Lasso) regression analysis and PPI analysis. Immune cell infiltration in MDD patients was analyzed using CIBERSORT, and correlation analysis was performed between key immune cells and genes. The diagnostic accuracy of the identified hub genes was evaluated using receiver operating characteristic (ROC) curve analysis. Furthermore, we conducted a study involving 10 MDD patients and 10 healthy controls (HCs) meeting specific criteria to assess the expression levels of these hub genes in their peripheral blood mononuclear cells (PBMCs). The Herbal Ingredient Target Database (HIT) was employed to screen for herbal components that target these genes, potentially identifying novel therapeutic agents.

**Results:**

A total of 159 down-regulated and 51 up-regulated genes were identified for further analysis. WGCNA revealed 12 co-expression modules, with modules “darked”, “darkurquoise” and “light yellow” showing significant positive associations with MDD. Functional enrichment pathway analysis indicated that these differential genes were associated with immune functions. Integration of differential and immune-related gene analysis identified 21 common genes. The Lasso algorithm confirmed 4 hub genes as potential biomarkers for MDD. GSEA analysis suggested that these genes may be involved in biological processes such as protein export, RNA degradation, and fc gamma r mediated cytotoxis. Pathway enrichment analysis identified three highly enriched immune-related pathways associated with the 4 hub genes. ROC curve analysis indicated that these hub genes possess good diagnostic value. Quantitative reverse transcription-polymerase chain reaction (RT-qPCR) demonstrated significant expression differences of these hub genes in PBMCs between MDD patients and HCs. Immune infiltration analysis revealed significant correlations between immune cells, including Mast cells resting, T cells CD8, NK cells resting, and Neutrophils, which were significantly correlated with the hub genes expression. HIT identified one herb target related to IL7R and 14 targets related to TLR2.

**Conclusions:**

The study identified four immune-related hub genes (TLR2, RETN, HP, and IL7R) in MDD that may impact the diagnosis and treatment of the disorder. By leveraging the GEO database, our findings contribute to the understanding of the relationship between MDD and immunity, presenting potential therapeutic targets.

## Introduction

Major depressive disorder (MDD) is a prevalent mental illness characterized by symptoms such as a depressed mood, diminished interest or pleasure in activities, reduced energy, and suicidal ideation (1). The incidence of depression is on the rise, with an estimated 300 million individuals globally affected by MDD (2). Projections indicate that depression may become the leading cause of the global burden of disease by 2030 (3). MDD is associated with a high disability rate, significant suicide risk, substantial societal burden, a tendency for recurrence, and a challenge in timely access to effective treatment for the majority of patients (4). These factors have positioned MDD as a significant public health and social issue, adversely impacting the physical and mental well-being of individuals. In contrast to the substantial patient population and disease burden, the current diagnostic landscape for mental illnesses is limited by the absence of effective, straightforward, and reliable objective diagnostic methods. This deficiency impedes the accurate diagnosis, treatment, and pathophysiological research of mental disorders. Consequently, the identification of diagnostic biomarkers has emerged as an urgent and critical priority, essential for facilitating precise diagnosis and treatment strategies.

Despite the large number of experimental and clinical studies conducted to elucidate the pathogenesis of depression, including genetic, biological, psychological, and social determinants, our understanding of the etiology of MDD remains incomplete (5, 6). Antidepressant medications available, 30-50% of patients with MDD do not achieve complete remission, suggesting that conventional treatments do not address important biological processes involved in MDD pathology (7). The genetic factors in the pathogenesis of MDD are complex and involve the interaction of multiple genes with environmental factors. Research has shown that MDD is polygenic, meaning that it is affected by the combined effects of multiple genes rather than a single gene. Rapid advances in sequencing technology and bioinformatics have made it possible to explore the disease pathogenesis at the gene level (8). One of the more important methods is weighted gene co-expression network analysis (WGCNA). This analysis method aims to find co-expressed gene modules and explore the association between the gene network and the phenotype of interest, as well as the core genes in the network (9). In this study, using abundant public resources and bioinformatics methods, the hub genes associated with depression were identified through differential analysis of the GEO database and WGCNA, respectively. Gene ontology (GO) and Kyoto Encyclopedia of Genes and Genomes (KEGG) were utilized to further investigate the biological processes and pathways. The functional enrichment pathway analysis revealed that these differential expressed genes (DEGs) were associated with immunity.

**Figure 1:**
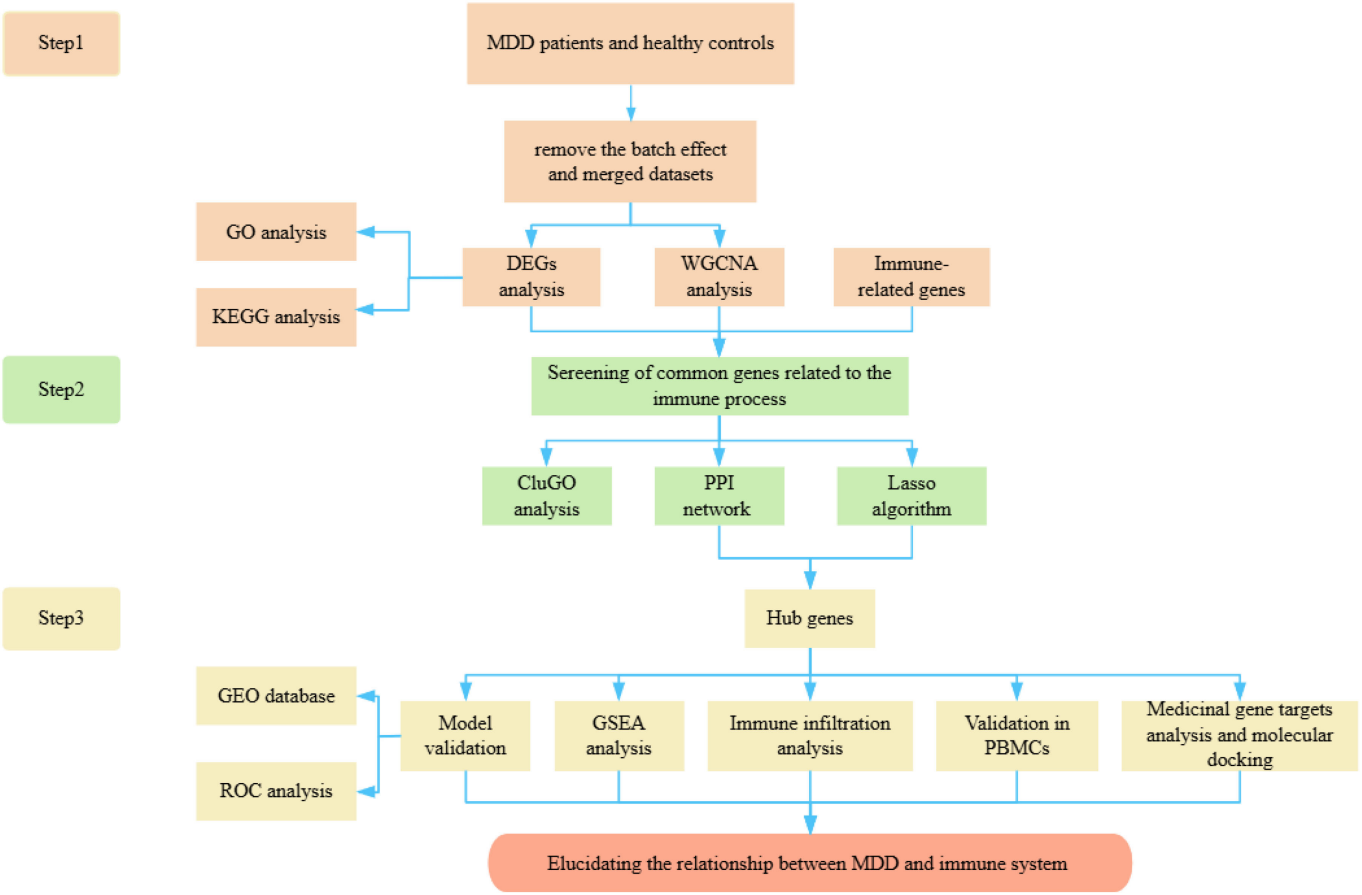
The fowchart of the study.

Evidence from numerous studies indicates that MDD is associated with immune dysregulation, including monocyte activation, reduced T-cell numbers and activity, and elevated pro-inflammatory cytokine production (10, 11). To understand the relationship between MDD and the immune system, we conducted a comprehensive analysis of DEGs in MDD, genes from WGCNA module, and immune-related genes using a Venn diagram approach. This analysis revealed 21 overlapping genes, which were further subjected to protein-protein interaction (PPI) network analysis and ClueGo analysis. Furthermore, the least absolute shrinkage and selection operator (Lasso) was used to further identify the key genes. Ultimately, we identified four hub genes, which were validated on three datasets, respectively, and an additional external verification set, GSE39653, was used for validation. The area under the curve (AUC) values indicated the superior diagnostic performance of the model, offering prospects for the diagnosis of MDD. Additionally, we investigated immune infiltration patterns and the correlation of various immune factors with the identified hub genes. The expression levels of these hub genes were examined in peripheral blood mononuclear cells (PBMCs), revealing significant differences. Finally, pathway analysis of the four hub genes showed that TLR2 and IL7R were more closely involved in the immune pathway, therefore, we conducted network pharmacological analysis and molecular docking specifically on these two genes. In conclusion, given that the pathogenesis of MDD remains ambiguous and an effective biomarker is lacking in clinical practice for the identification and accurate treatment of MDD. In this study, immune-based hub genes and drug targets were identified through diverse bioinformatics approaches, which are anticipated to guide the diagnosis and enable accurate treatment in MDD patients. This study enriches our understanding of the pathogenesis of psychiatric disorders and provides a systematic framework for comprehending the molecular basis of MDD, offering novel insights into its diagnosis and treatment. The workflow of this study is presented in **Figure 1**.

## Materials and methods

### Subject recruitment

The research protocols were approved by the medical ethics committee of the Third Hospital of Shanxi Medical University. All participants voluntarily participated in the study and provided informed consent. Two licensed and experienced psychiatrists were responsible for the recruitment process. Each procedure adhered to applicable rules and regulations. The Diagnostic and Statistical Manual of Mental Disorders (5th edition) and the Hamilton Depression Rating Scale-24 items (HAMD-24) were used to determine the diagnosis of patients with MDD. Exclusion criteria included individuals with co-existing physical or mental conditions or substance abuse. In addition, 10 healthy controls (HCs), none of whom had neurological or systemic medical conditions or DSM-IV Axis I/II disorders, were recruited from the same hospital’s medical screening center during the same period. The demographic and clinical characteristics of the included patients are shown in **Table 1**.

**Table 1:** Demographic characteristics of MDD patients and HCs.

Variables	MDD patients (n=10)	Healthy controls (n=10)	*P-value* ^a^
Age (year)^b^	34.8 ± 13	32.6 ± 9.6	0.724
BMI (kg/m²)^b^	21.9 ± 2.7	22.6 ± 3.5	0.582
Gender (M/F)	4/6	4/6	1.000
HAMD-24 total score^a^	25.1 ± 4.3	1.7 ± 1	<0.001
Antidepressants	10 (100%)	–	–
Antipsychotics	6 (60%)	–	–
Mood stabilizers	3(30%)	–	–

MDD, major depressive disorder; HCs, health controls; M/F, male/female; BMI, Body Mass Index.

a two independent samples t-test for continuous variables (age and BMI); mann whitney wilcoxon test for categorical variables (sex).

b Values expressed as means ± SDs.

### Data acquisition and processing

Gene expression data of MDD and their clinical details were obtained from the GEO databases (https://www.ncbi.nlm.nih.gov/geo/) specifically GSE32280, GSE76826, GSE98793, GSE39653. GSE32280 contains information on postmortem brain tissue samples from 16 MDD patients and 8 matched HCs. All tissue samples were based on the platform of GPL570, which enabled the transformation of probes into corresponding gene symbols. GSE76826, annotated by GPL17077, included 10 MDD patients and 12 HCs, while GSE98793, annotated by GPL570, included 126 MDD patients and 64 HCs. To enhance the credibility and comprehensiveness of the results, which can be better assessed for their clinical relevance and potential prognostic value, we chose GSE39653 as the verification cohort, annotated by GPL10558, included 21 MDD patients and 24 HCs. We utilized the R package “inSilicoMerging” to eliminate the batch effects and merged the GSE32280, GSE76826 and GSE98793 datasets into a single set. After consolidating the data, we obtained a sample of 154 MDD patients and 84 HCs. **Table 2** provides details of the dataset.

**Table 2:** Information of GEO datasets containing the MDD patients and HCs.

ID	GSE number	Platform	Samples
1	GSE32280	GPL570	16 MDD patients and 8 HCs
2	GSE76826	GPL17077	10 MDD patients and 12 HCs
3	GSE98793	GPL570	128 MDD patients and 64 HCs
4	GSE39653	GPL10558	21 MDD patients and 24 HCs

In addition, we searched for the term “immune” in the gene card (https://www.genecards.org/) to retrieve genes related to the immune system. Genes with an association score of > 7 were considered to be immune-associated (**Supplementary Table S1**).

### Differential expression analysis

Differential expression analysis among MDD clusters was identified using the “limma” package in R software, and DEGs were screened based on the criterion (P < 0.05 and 1.15 fold change). Heatmaps and volcano plots of the DEGs were created using the “pheatmap” and “ggplot2” packages.

### Weighted gene co-expression network analysis

To identify important modules and key genes in the MDD clusters, a gene co-expression network was constructed using the “WGCNA” package in R software (12). Firstly, we calculated the MAD (Median Absolute Deviation) of each gene by using the gene expression profile, and eliminated the top 50% of genes with the smallest MAD, and removed outliers and samples by using the goodSamplesGenes method of the R software package WGCNA. Then, a scale-free representation network was created using the weighted adjacency matrix and the soft threshold parameter β. The soft threshold parameter β is used to adjust the connection strength of the network to ensure that the network structure conforms to the characteristics of scale-free networks. The adjacency matrix was then converted into the topological overlap matrix (TOM). Based on the TOM-based difference metric (1 - TOM), these genes were hierarchically clustered using the flashClust function. After hierarchical clustering, highly interconnected genes were assigned to the same module. The minimum number of genes per module was set to 30. In addition, we merged modules with a distance of less than 0.25 and finally obtained 12 co-expression modules. It is worth noting that the grey module is considered a gene set that cannot be assigned to any module.

### Enrichment analysis

In order to further understand the biological processes and signaling pathway regulation involved in DEGs, we performed GO and KEGG analyses on the corresponding genes. GO is widely used for large-scale functional enrichment analyses, including biological processes (BP), molecular functions (MF), and cellular components (CC). KEGG is a well-known reference database that contains information on genomes, biological pathways, diseases, and drugs (13). The threshold value of *P-value* was 0.05. In addition, to further explore the relationship between MDD and immune, we applied the ClueGO packages to the genes for functional enrichment analysis, so that functionally related genes formed a network and a cluster form of the diagrams.

### Construction of protein-protein interaction network and key genes selection

The Venn diagram package in R was utilized to identify overlapping genes associated with MDD and immunity by taking the intersection of DEGs, immune genes, and modular genes obtained from WGCNA. Subsequently, in order to systematically analyze the biological functions of the overlapping genes, we located these genes in the STRING database, a tool for predicting interactions between genes or proteins (14). A PPI network was constructed using the results of PPI pairs interaction scores > 0.4. In the PPI network, there are 20 nodes and 42 edges, with a PPI enrichment *P-value* of 1.11e-16. Subsequently, the PPI network was visualized by Cytoscape software (version 3.9.0) (15). To identify key proteins or hub nodes in the PPI network, we created a key subnetwork in CytoNCA by computing the topological parameters of each node in the network and setting the filtering threshold for secondary filtering (16). Finally, CytoHubba was used to identify key genes by applying multiple topological network algorithms and ranking node genes in the network. The top 10 genes were eventually identified for the next step of analysis.

### Screening characteristic genes via machine learning

In order to reduce the number of genes in the model and to solve the problem of multicollinearity in the regression analysis, we used Lasso regression analysis for feature selection and screening of diagnostic markers for MDD (17). The Lasso algorithm was implemented using the “glmnet” software package, and the candidate genes in the last step were further screened through Lasso analysis.

### Validation of the diagnostic ability of the hub genes

In this study, we employed the R package “glmnet” to integrate survival time, survival status, and gene expression data for regression analysis utilizing the lasso-Cox method. The Lambda value was set to 0.0317286995003536. Furthermore, we implemented a 10-fold cross-validation procedure to identify the optimal model. The “pROC” R package was used to calculate the AUC of the receiver operating characteristic (ROC) curves to assess the predictive effect of the feature genes in the training and validation sets. The AUC value is generally between 0.5 and 1, and the closer to 1, the better the diagnosis effect.

### Immune infiltration and immune-related factors

Immune cell infiltration in the microenvironment was assessed using CIBERSORT, which contains 22 human immune cells, including plasma, B cell, T cell, and myeloid cell subpopulations. The tool is based on the principle of linear support vector regression, which analyzes the expression matrix of immune cells by deconvolution analysis. The abundance of 22 immune cell phenotypes in these samples was predicted by uploading the expression matrix data from GSE32280, GSE76826, GSE98793. Then, we used the “ggplot2” package to draw violin plots to visualize differences in immune cell infiltration between MDD patients and HCs samples. We also calculated Spearman correlations between identified hub genes and immune cells.

### Gene set enrichment analysis

To better understand the biology and pathways of hub genes, we used GSEA to determine the distribution of predefined gene sets in a phenotype-based gene ordering table and to assess their impact on phenotype (18). We obtained the GSEA software (version 3.0) from the GSEA website (http://software.broadinstitute.org/gsea/index.jsp). According to the expression level of genes, samples were divided into a high expression group (≥50%) and a low expression group (<50%). The minimum gene set was set at 5 and the maximum gene set at 5000, with 1000 resampling. *P value* < 0.05 and FDR < 0.25 were considered statistically significant.

### Quantitative reverse transcription-PCR

Venous blood was collected from all participants using a blood collection tube containing ethylenediaminetetraacetic acid (EDTA). Peripheral blood mononuclear cells (PBMCs) were subsequently isolated from whole blood samples using Ficoll solution (Solarbio Life Sciences, China). Total RNA was extracted from the PBMCs employing the TransZol Up Plus RNA Kit (TransGen, China). RNA quality was assessed using a NanoVue Plus spectrophotometer (Biochrom, UK), followed by RT-qPCR on a CFX96 real-time PCR detection system (Bio-Rad, USA). The expression of GADPH encoding 3-phosphoglyceraldehyde dehydrogenase was used as a reference for data normalization. The list of primers used in the RT-qPCR experiment is shown in **Table 3**. The fold changes of the indicated genes were calculated using the 2^-ΔΔCt^ method.

**Table 3:** RT-qPCR primers.

Primers	Sequences (5’-3’)
TLR2	Forward: CCCCTAACAGAGATTTACCCA
	Reverse: TAAGCCACAACAATAGCAT
RETN	Forward: TATGCCCACAGGGACCTAGC
	Reverse: CAGTCTCCTGCACTCACCTCG
HP	Forward: GCCCCTACTTTAACACTC
	Reverse: CAGCCAGAAACATAACCCA
IL7R	Forward: AGCCAATGACTTTGTGGTGAC
	Reverse: GGCCCTCCCAGGGGAAATGC
GADPH	Forward: CAACAGCCTCAAGATCATCAGGT
	Reverse: CATACCATGAGTCCTTCCACGAT

### Statistical analysis

All analyses were performed in R software. The t-test and Mann-Whitney U test were chosen based on whether the data conformed to a normal distribution. Significance was usually defined as *P-value* < 0.05.

### Screening of medicinal gene targets and drugs

As a rich source of candidate drugs, herbal active ingredients play a vital role in the development of new drugs. To better understand the interactions between herbal compounds and molecular targets, the first herbal ingredients’ targets database (HIT) (http://lifecenter.sgst.cn/hit/) (19) was established, which can be easily linked to therapeutic targets database (TTD) and drug library, etc. We used HIT to screen herb ingredients’ targets for hub genes to determine possible therapeutic targets.

## Results

### Information of included GEO datasets

**Figure 2:**
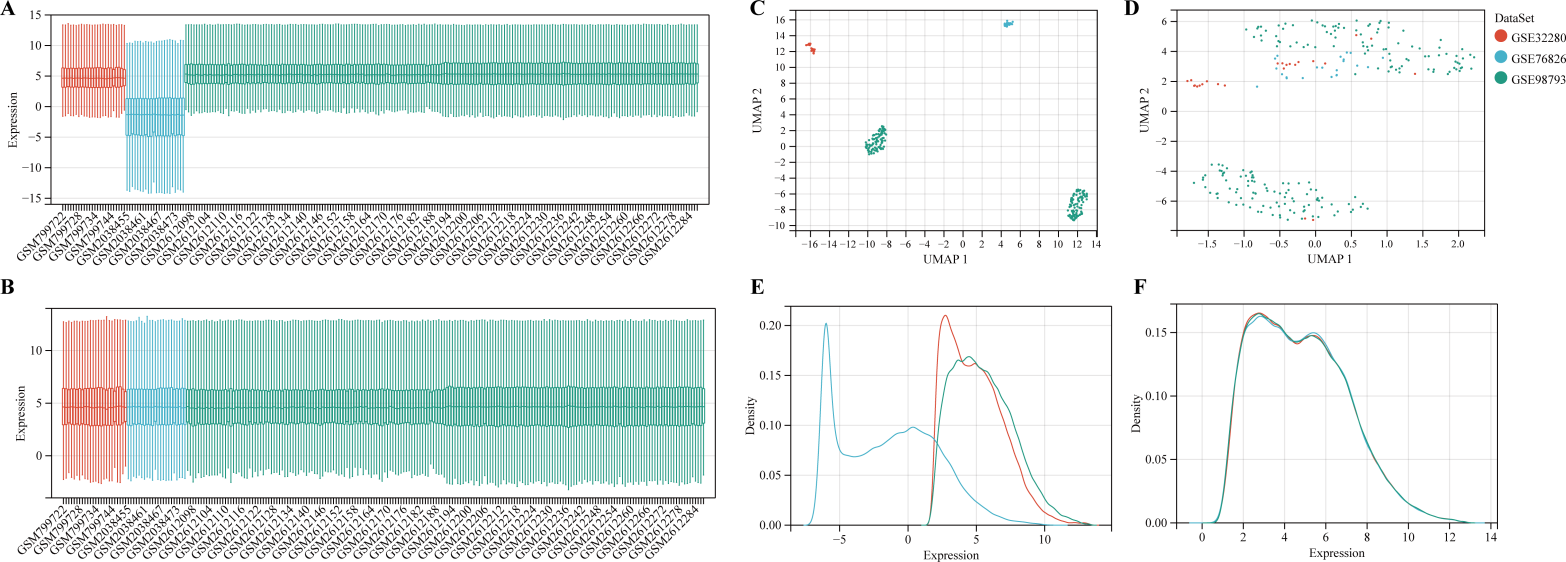
Data preprocessing. **(A, B)** The boxplot of the merged microarray datasets before and after normalization. **(C, D)** UMAP plots of the merged microarray dataset before and after normalization. **(E, F)** Density plots of the merged microarray dataset before and after normalization.

According to the previously established inclusion criteria, GSE32280, GSE76826 and GSE98793 were included in this study. There were 154 MDD patients and 84 HCs in these three datasets. In order to eliminate the bath effect from different platforms and batches, we eliminated the batch effect from three datasets and then merging different platforms (**Figure 2**). From the box plots and density plots, we observed that before removing the batch effect, the sample distribution of each dataset varied greatly, suggesting that there was a batch effect. After removing the batch effect, the distribution of the data between each dataset tends to be the same, indicating that the batch effect is better removed.

### Identification of DEGs in MDD patients

**Figure 3:**
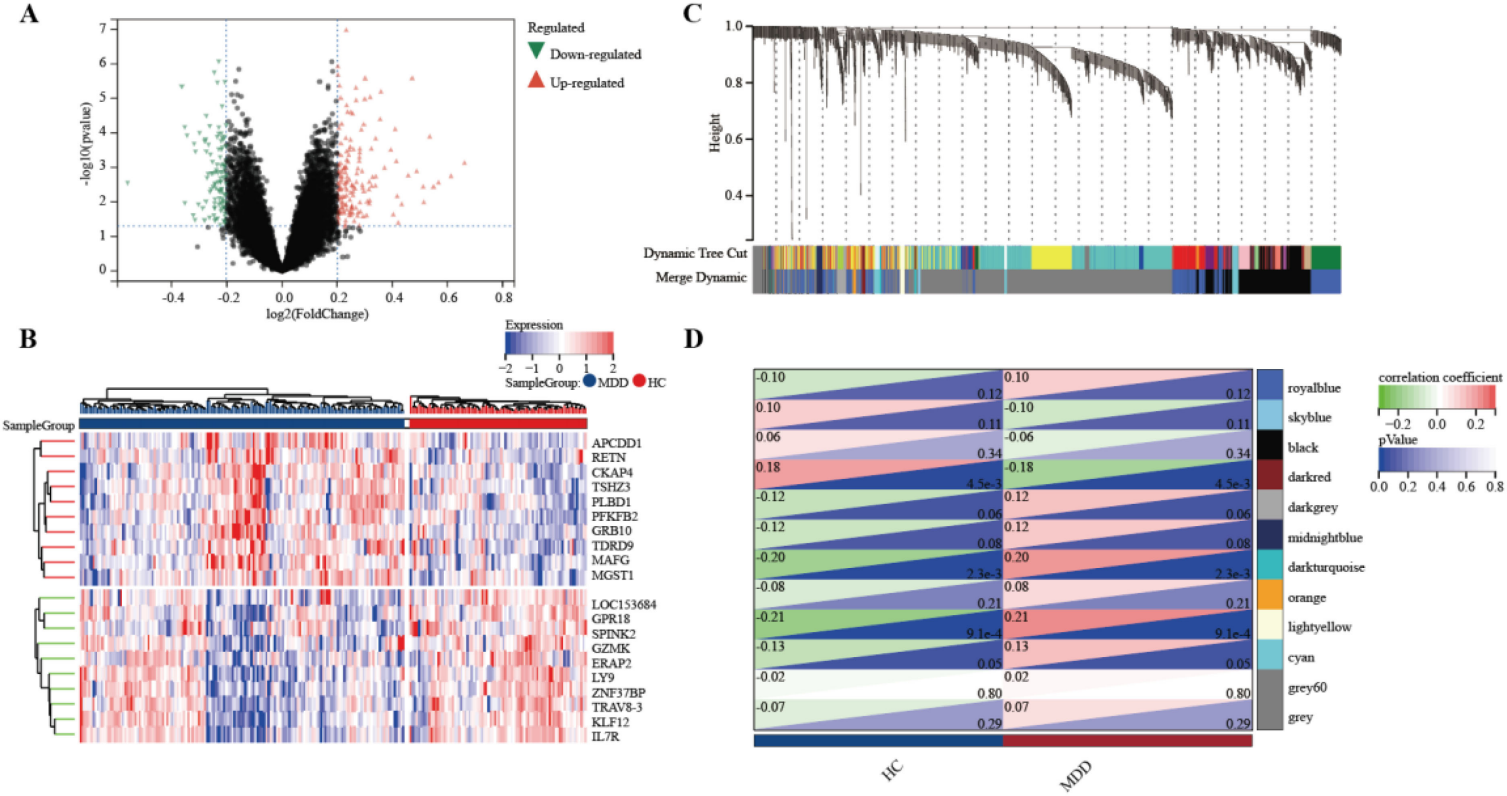
Determine the intersection genes of WGCNA and DEGs. **(A)** Volcano plot of DEGs. Red genes represent up-regulated genes; Green genes represent down-regulated genes; Black genes represent unchanged genes. **(B)** The heatmap shows the top 10 genes significantly highly expressed in MDD patients or HCs. Red genes represent higher expression; blue genes represent lower expression. **(C)** Gene dendrogram with clustering. **(D)** Module-trait relationship heatmap.

To identify genes associated with MDD, we first obtained 263 differentially expressed genes from GSE32280, GSE76826, and GSE98793 using the screening condition “*P-value* < 0.05, 1.15 fold change”. These DEGs are shown in the volcano plot (**Figure 3A**), including 159 up-regulated genes and 104 down-regulated genes. A heatmap of the top 10 differentially expressed genes was drawn (**Figure 3B**). The details of DEGs expression are in **Supplementary Table S2**. We identified 12 modules in three datasets by WGCNA, with each module represented by a different color. Based on the Spearman correlation coefficient a, a heatmap of module-trait relationships was drawn to assess the relationships between modules (**Figures 3C, D**). Three modules “darked”, “darkurquoise”, and “light yellow” had a highly positive association with MDD, which were chosen as MDD-related modules (darked module: r = 0.18 p = 4.5e-3; darkurquoise module: r = 0.20, p = 2.3e-3; light yellow module: r = 0.21, p = 9.1e-4). The module genes identified in the WGCNA can be found in **Supplementary Table S3**. The number of genes in each module is shown in **Supplementary Table S4**.

### GO and KEGG analysis

**Figure 4:**
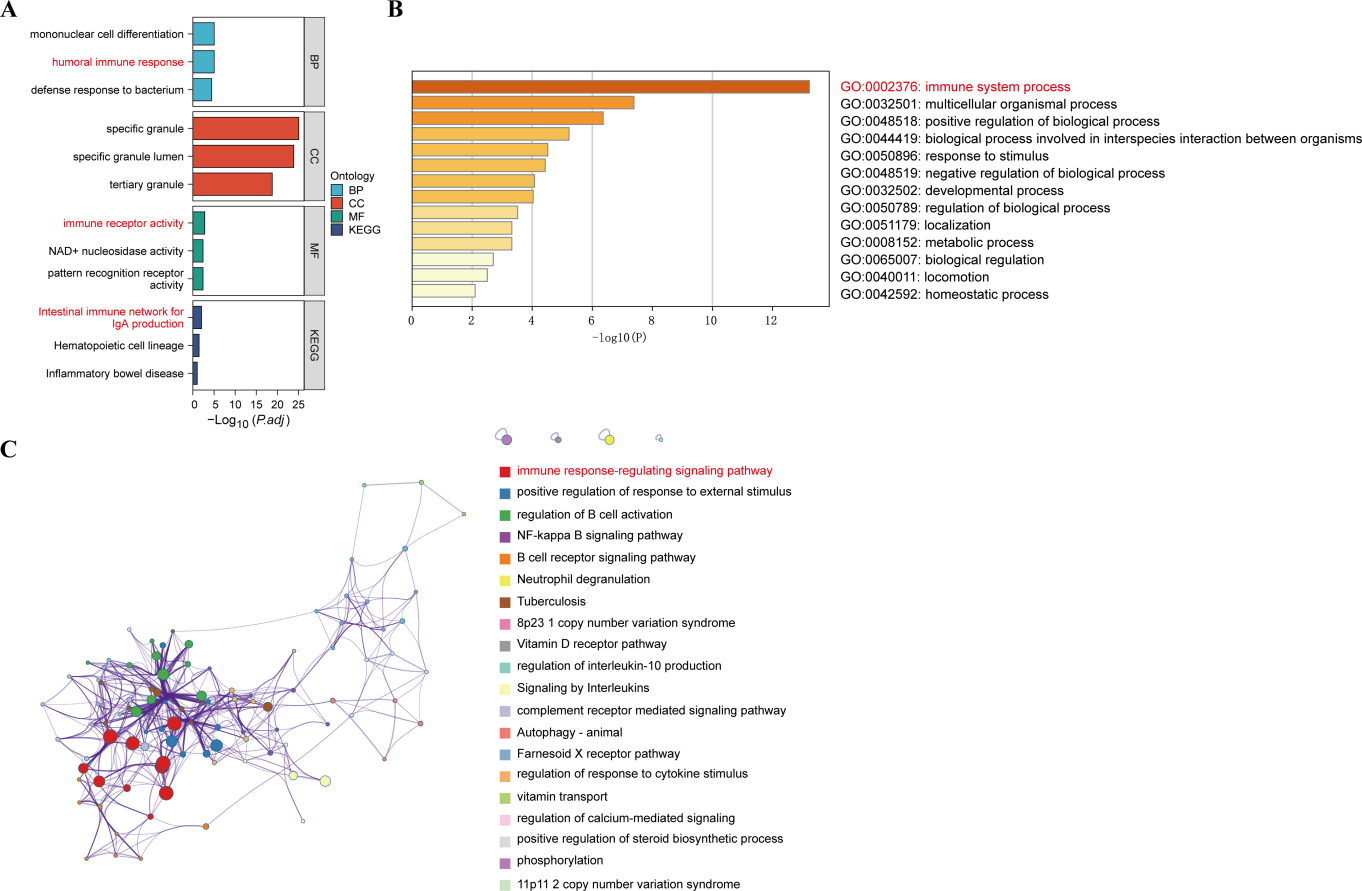
GO functional and KEGG pathway analysis of DEGs. **(A)** GO biological process of the four clusters. **(B)** Heatmaps of enriched terms in differentially expressed gene lists were entered via metscape and coloured with *P-values*. **(C)** Visualisation of the metascape protein-protein interactions enrichment networks illustrating intra- and inter-cluster similarity of enriched phrases, with cluster annotations being colour-coded.

To understand the potential enrichment pathways associated with DEGs, we performed GO and KEGG pathway enrichment analyses. In terms of biological processes, the GO enrichment analysis of MDD revealed that these target genes were primarily associated with the humoral immune response (**Figure 4A**). Regarding cellular components, the target genes exhibited significant enrichment in specific granule and tertiary granules. In the realm of molecular function, the target genes were significantly enriched in immune receptor activity. These target genes also demonstrated enrichment in KEGG pathways such as the Intestinal immune network for IgA production, Hematopoietic cell lineage, and Inflammatory bowel disease. In addition, functional enrichment analysis using Metascape revealed that these genes were significantly enriched in immune system process, which correlated with MDD (**Figure 4B**). Functional enrichment of the genes in the WGCNA module showed that these genes were concentrated in the immune response-regulating signaling pathway (**Figure 4C**). These enriched pathways and terms significantly enhance our understanding of the role played by DEGs in the onset and progression of MDD.

### PPI network construction

**Figure 5:**
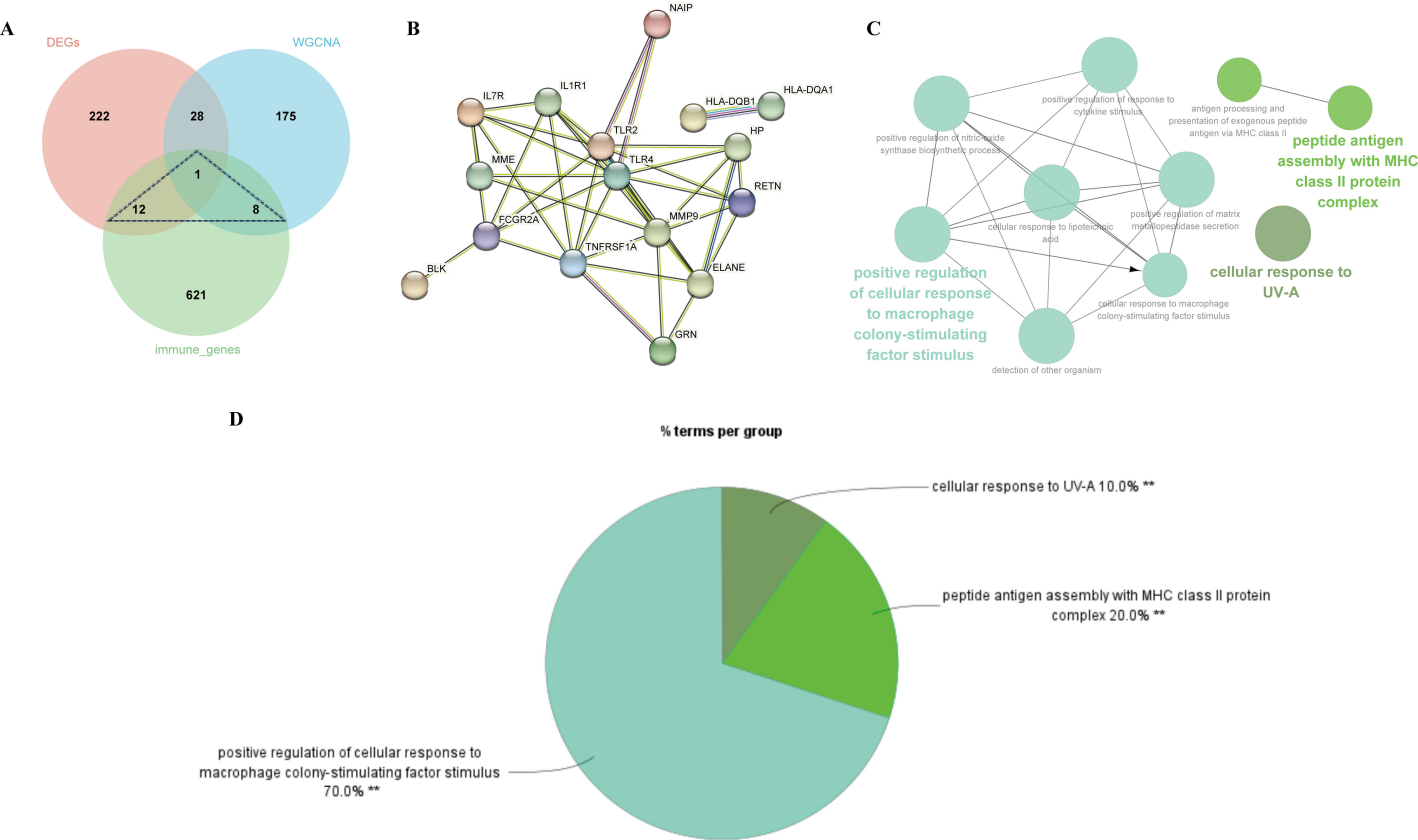
Identification of shared genes and construction of PPI network of the shared genes. **(A)** As shown by the venn diagram, the DEGs of MDD, module genes of the WGCNA and the immune genes were intersected in order to screen the target key genes. **(B)** PPI interaction network constructed from candidate key genes. **(C)** The network of GO terms in ClueGO. **(D)** The percentage of GO terms in the shared genes.

By extracting crossover genes from 263 DEGs, 212 modular genes, and 642 immune-related genes, we succeeded in identifying 21 target genes associated with MDD and immune-related genes (**Figure 5A**). In this study, the STRING database was used to construct PPI networks associated with 21 crossover genes (**Figure 5B**). To investigate the potential role of shared genes, we performed GO analysis using ClueGO, which showed that 70% of these genes were concentrated in the positive regulation of cellular response to macrophage colony-stimulating factor stimulus, suggesting that this pathway is important in MDD and immunity. 20% on peptide antigen assembly with the MHC class II protein complex, and 10% on cellular response to UV-A (**Figure 5C, D**). Detailed findings from the ClueGO analysis can be found in **Supplementary Table S5**.

### Screening and validation of diagnostic genes in patients with MDD

**Figure 6:**
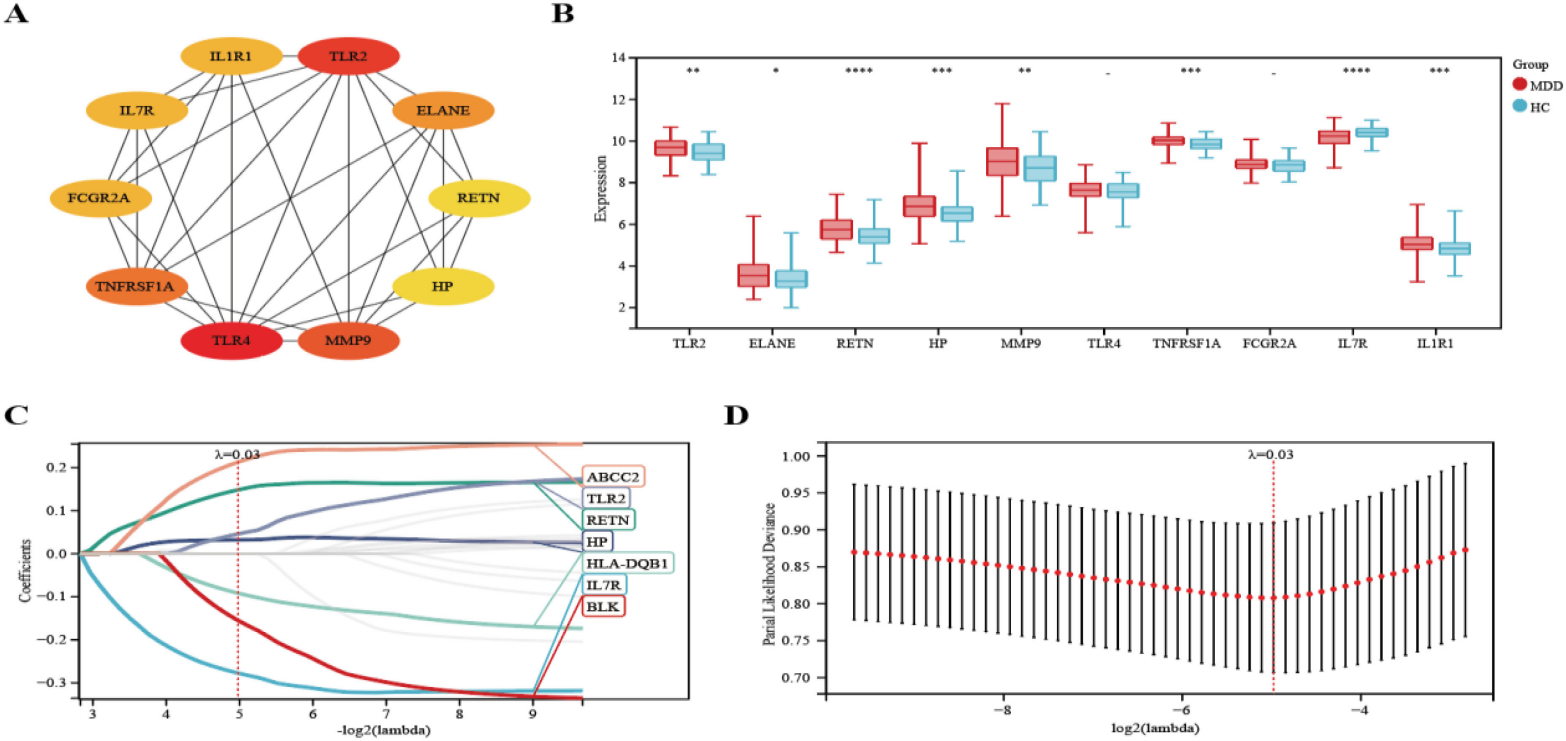
Lasso coefficient profiles of the top 10 genes in PPI network. **(A)** The PPI network’s top 10 genes are shown, ranked from red (high degree value) to yellow (low degree value), in order. **(B)** Differential expression analysis of 10 genes. **(C)** The trajectory of the independent variable with the change of lambda. **(D)** Lasso regression under each Lambda confidence interval. Significant differences were supposed at *p-value < 0.05, **p-value < 0.01, ***p-value < 0.001, **** p-value < 0.0001, compared with the control.

We selected the top ten genes from 21 target genes based on Cytohubba analysis (**Figure 6A**). We then analyzed the expression of these genes in MDD patients and HCs and found that eight genes were significantly different (TLR2, ELANE, RETN, HP, MMP9, TNFRSF1A, IL7R, IL1R1) (*P-value* < 0.05) (**Figure 6B**). In addition, we used the Lasso algorithm to screen for 7 characterized genes (ABCC2, TLR2, RETN, HP, HLA-DQB1, IL7R, BLK) from 21 genes (**Figures 6C, D**).

### Diagnostic model based on four hub genes

**Figure 7:**
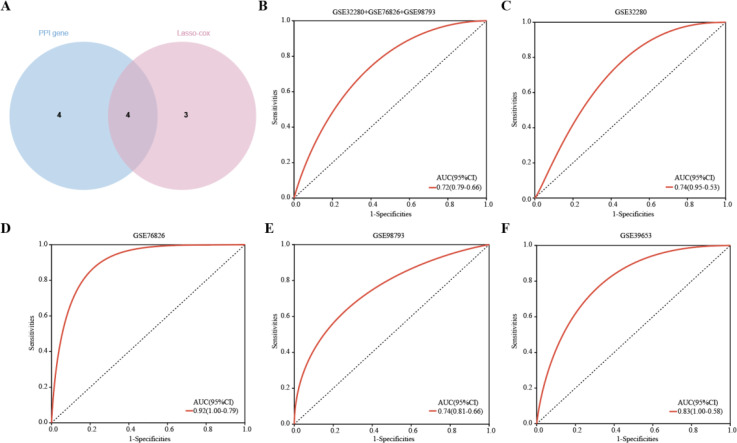
Hub genes were identified and ROC analysis was used to evaluate the prediction efficiency. **(A)**Venn diagrams showing the overlap of the hub genes in the PPI network and Lasso-cox. **(B-F)** ROC curve analysis of the MDD diagnostic model. ROC is verified for three datasets respectively, and another external verification set GSE39653 is verified.

Four hub genes, identified by extracting eight significantly expressed genes from the PPI network and seven characterized genes using Lasso-Cox, were TLR2, RETN, HP, and IL7R. (**Figure 7A**). Details of these 4 hub genes are shown in **Table 4**. Based on three datasets of MDD, we developed a diagnostic prediction model using Lasso regression analysis. GSE32280, GSE76826, and GSE98793 were used for internal validation of the model, and the AUC values were 0.74, 0.92, and 0.74, respectively. The AUC of the three datasets combined into one dataset was 0.72 (**Figures 7B–E**). In addition, diagnostic prediction models were evaluated using the external verification set GSE39653. The AUC of the diagnostic model was 0.83, indicating good diagnostic performance (**Figure 7F**). Based on this information, the 4 hub genes described above may be sensitive and specific for distinguishing MDD samples from normal samples, suggesting that the model could guide the diagnosis of patients with MDD in clinical applications.

**Table 4:** Details of the four hub genes.

Gene Abbreviation	Gene Name	*p value*	Log FC
TLR2	toll like receptor 2	0.001425168	0.206093712
RETN	resistin	6.21E-06	0.35934477
HP	haptoglobin	7.60E-05	0.410088526
IL7R	interleukin 7 receptor	3.60E-06	-0.23387795

### Enrichment analysis of GSEA

**Figure 8:**
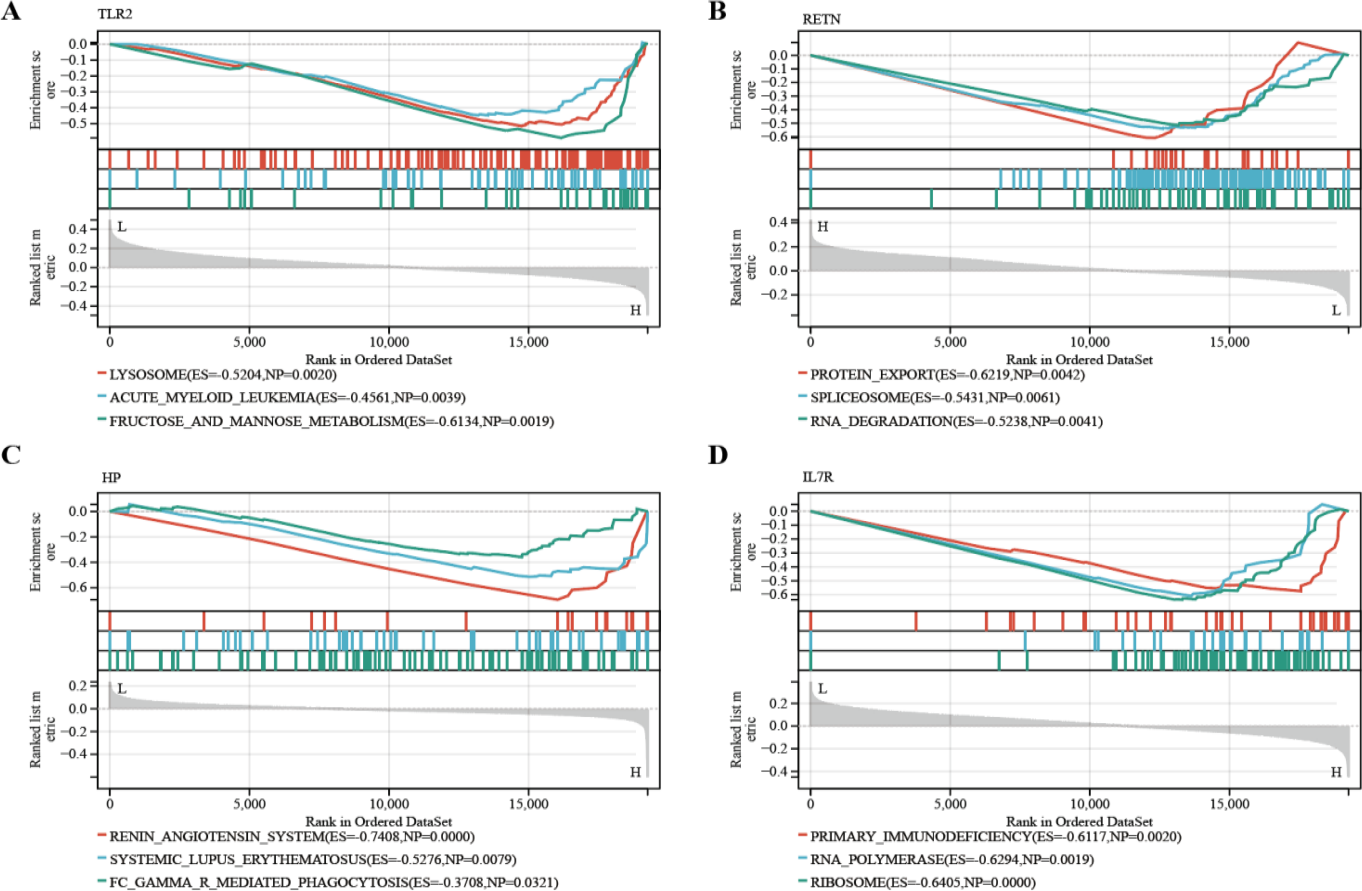
GSEA identified the four hub genes’ enriched pathways. **(A)** TLR2 **(B)** RETN **(C)** HP **(D)** IL7R.

GSEA was then performed to explore the molecular functions of these hub genes, and the enriched pathways of hub genes were shown in the **Figures 8A–D**. The results showed an association with several immune disorders, namely acute myeloid leukemia (ES=0.4561, NP=0.0039), systemic lupus erythematosus (ES=0.5276, NP=0.0079) and primary immunodeficiency (ES=0.6117, NP=0.0020). The possible involvement of these genes in disease development involves biological processes such as protein export (ES=0.6219, NP=0.0042), RNA degradation (ES=0.5238, NP=0.0041) and FC gamma r mediated phagocytosis (ES=0.3708, NP=0.0321).

### Expression validation of hub genes by RT-qPCR

**Figure 9:**
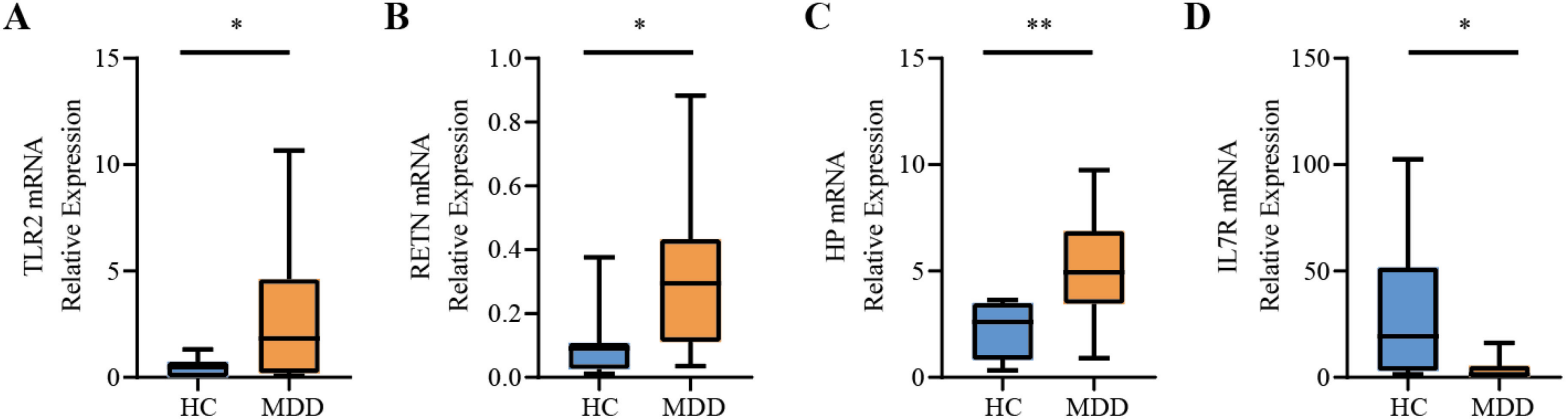
Relative expression levels of the four hub genes by RT-qPCR analysis. **(A)** TLR2. **(B)** RETN. **(C)** HP. **(D)** IL7R. Compared with the control group, it is considered that there are significant differences when the *P-value < 0.05, **P-value < 0.01.

To more comprehensively assess the levels of TLR2, RETN, HP, and IL7R in MDD, we analyzed the mRNA expression of these hub genes in PBMCs from healthy individuals and patients with MDD using RT-qPCR. The primer information of RT-qPCR is shown in **Table 3**. IL7R expression was significantly down-regulated in cells from MDD patients compared to normal cells (*P-value* < 0.05), and TLR2, RETN, and HP were significantly up-regulated in cells from MDD patients. The expression patterns matched the datasets for each disease (**Figure 9**).

### Immune infiltration and immune-related factors

**Figure 10:**
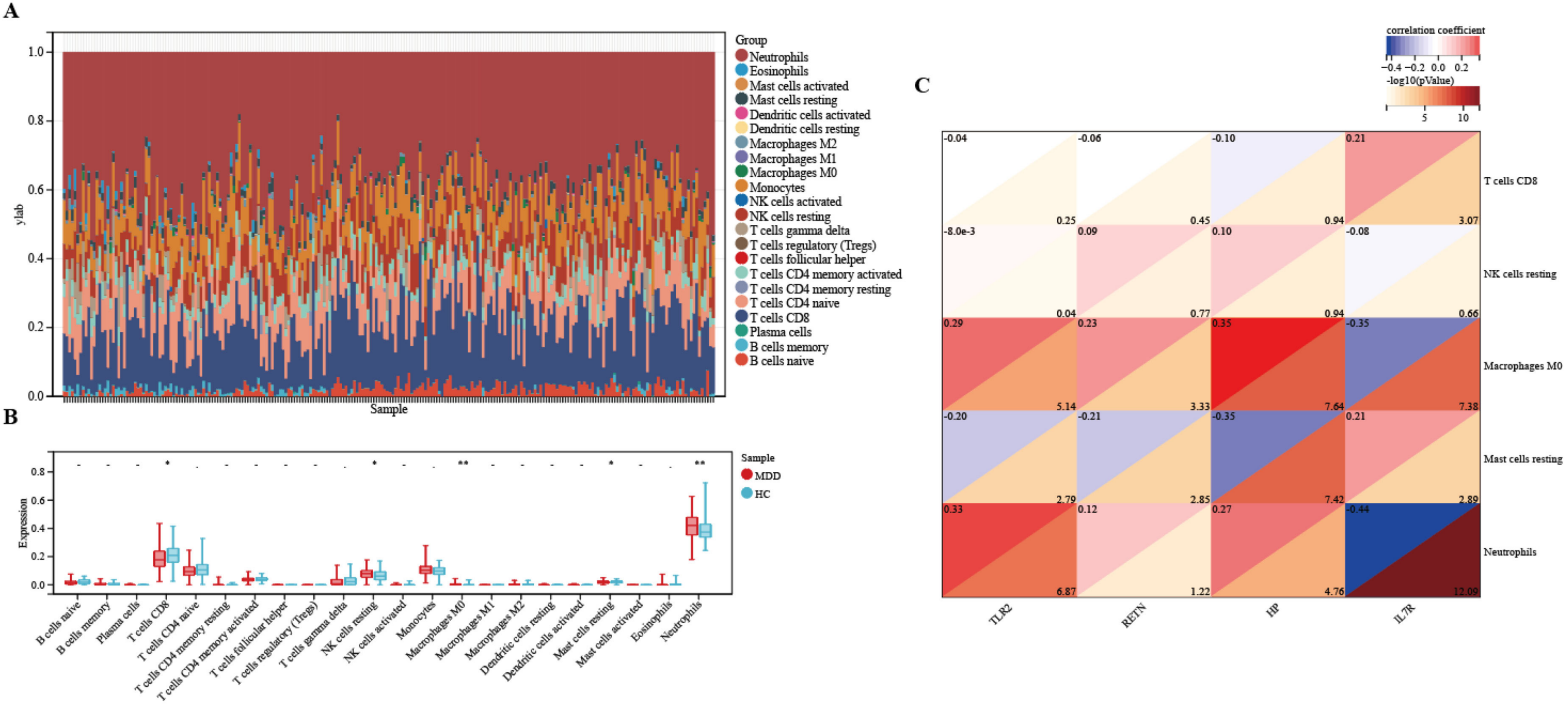
Immune infiltration between MDD and HCs. **(A)** The relative percentage of 22 immune cells in each sample. **(B)** Differences in immune infiltration between MDD and HCs samples. **(C)** Correlation of hub genes with immune cells and different immune factors. Blue represents a positive correlation, and red represents a negative correlation. The darker the color is, the stronger correlation is. Significant differences were supposed at *p-value < 0.05, **p-value < 0.01, compared with the control.

The microenvironment consists of immune cells, extracellular matrix, inflammatory factors, and various growth factors, which have a significant impact on clinical treatment sensitivity and disease diagnosis. As mentioned earlier, for MDD, the pathway is enriched in the immune direction. Therefore, in this study, the CIBERSORT algorithm was used to estimate the proportion of 22 immune cells in 154 MDD samples and 84 HCs samples (**Figure 10A**). The immune cell infiltration in MDD and HCs samples was compared in a box line plot (**Figure 10B**). The results showed that the proportions of NK cell resting, Neutrophils and Macrophages M0 were higher in MDD patients than in HCs, whereas the proportions of T cells CD8 and Mast cell resting were relatively low (*P-value* < 0.05). After that, we wanted to further understand the correlation between the hub genes and these significantly different immune cells. After analysis, only TLR2 had the strongest negative correlation with NK cells resting (r = -8.0e-3, *P-value* = 0.04), which showed a significant difference. See **Figure 10C** for detailed results. These results suggest that hub genes play an important role in the immune microenvironment.

### Medicinal targets and drug screening results

**Figure 11:**
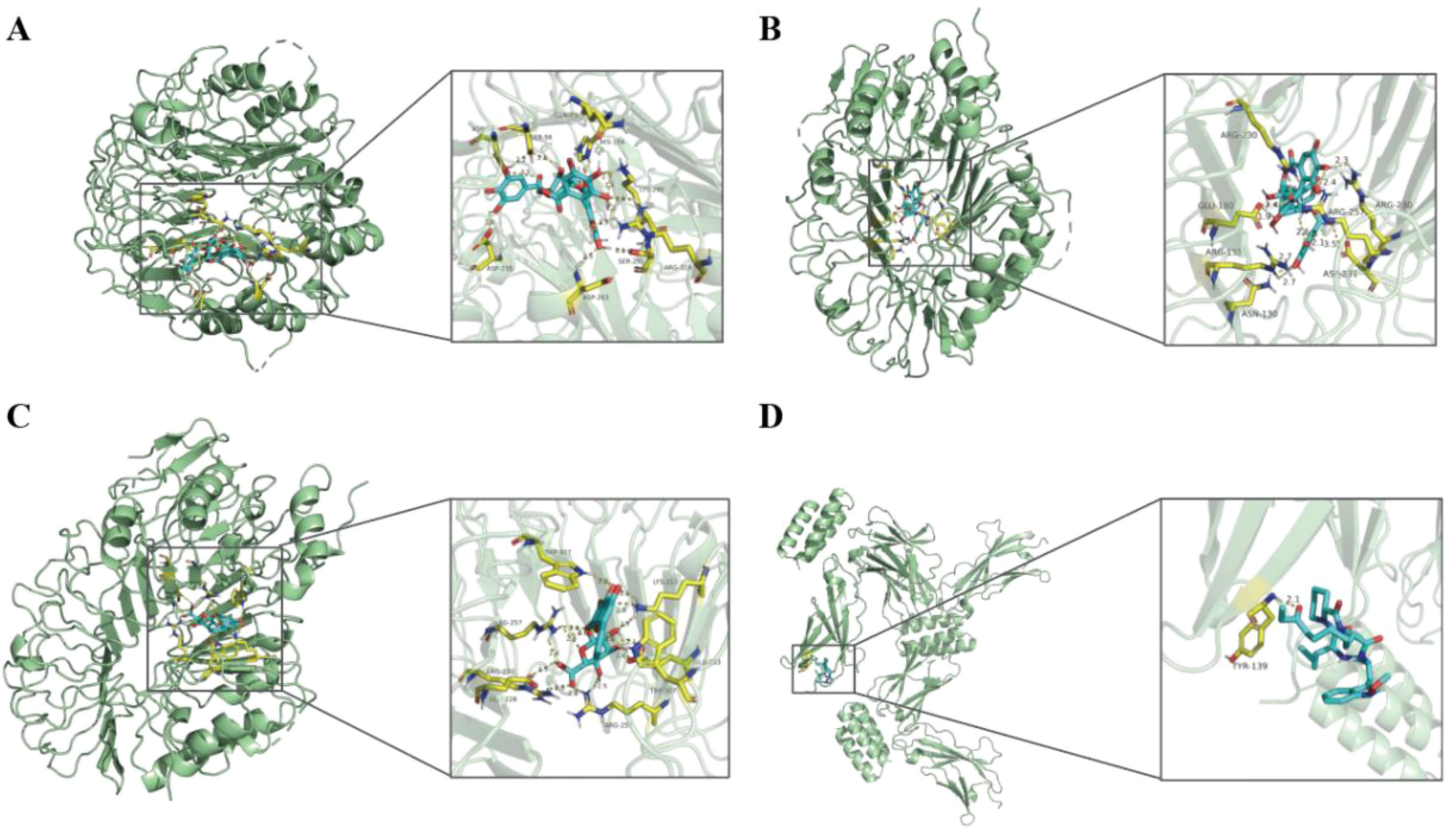
The results of molecular docking pattern of TLR2 with Corilagin (C0832) **(A)**, Corilagin (C1252) **(B)**, Baicalin (C0721) **(C)** with the top three binding energy, respectively. Molecular docking pattern of IL7R with Apicidin (C0803) **(D)**.

Pathways of four hub genes were analyzed, and it was found that these pathways with significance were concentrated in two genes, TLR2 and IL7R (**Supplementary Table S6**), such as the PI3K-Akt signaling pathway, the Toll-like receptor signaling pathway and the JAK-STAT signaling pathway, all of which were statistically significant (*P-value* < 0.05). Most of the enrichment pathways are immune-related. Therefore, we used HIT to screen herb compounds of TLR2 and IL7R, and found that TLR2 has 14 compounds, while IL7R has only one compound (**Supplementary Table S7**). Detailed herbal compound structures are shown in **Supplementary Figure S1**. Then TLR2 and IL7R were docked with their respective herbal compounds by molecular docking, detailed results are shown in **Supplementary Figure S2**. The top three herbal ingredients with binding energy related to TLR2 are Corilagin (C0832) (-11.8 kcal/mol), Corilagin (C1252) (-10.2 kcal/mol), and Baicalin (C0721) (-9.8 kcal/mol), respectively. The pharmaceutical compounds related to IL7R is Apicidin (C0803) (-3.4 kcal/mol) (**Figure 11**).

## Discussion

MDD is a highly heterogeneous disease characterized by high morbidity and mortality and is considered the most prevalent cause of disability worldwide (1). However, the pathophysiologic mechanisms of MDD are unknown, and the biomarkers used for diagnosis are unclear. In this study, we first scrutinized the RNA expression data of MDD patients and HCs to screen for DEGs. GO, KEGG enrichment analyses showed that DEGs were mainly concentrated in immune-related pathways. To explore the profound influence of immunity on the pathogenesis of MDD more deeply, we combined multiple bioinformatics approaches to identify four hub genes (TLR2, RETN, HP, and IL7R) as key biomarkers of MDD, they showed significant differential expression between MDD patients and HCs. In addition, we used HIT to screen the herbal compounds of TLR2 and IL7R, and performed molecular docking with their respective herbal compounds to identify three herbal ingredients, Apicidin, Corilagin and Baicalin, which may be used as antidepressants in the future to treat MDD patients. Importantly, the identification of such biomarkers will enhance preventive interventions and provide valuable insights into patients’ responses to antidepressant treatments. This advancement will further facilitate the monitoring of treatment effectiveness in patients. Consequently, the development of specific depression-related biomarkers is essential for diagnosing and tracking individuals with depression, potentially leading to earlier diagnosis and timely intervention, thereby significantly improving patient outcomes (20).

GO enrichment analysis showed that the DEGs screened from the merged dataset were mainly related to humoral immune response, specific granule, and tertiary granule, and immune receptor activity. A large genome-wide transcription study in the United Kingdom found that genes aberrantly expressed in MDD are involved in the regulation and implementation of the innate immune response (21). Another study suggests that humoral immune response may be a key link between brain and blood in MDD (22). A study demonstrated a clear association between the CD300f immune receptor and MDD in females, with CD300f-/- mice exhibiting several characteristic MDD features, such as an increased number of microglia and increased interleukin 6 and interleukin 1 receptor antagonist messenger RNA, as well as persistent depressive-like and pleasure-deprived behaviors (23). According to KEGG pathways analysis, DEGs were abundantly enriched in the IgA production, hematopoietic cell lineage, and inflammatory bowel disease. As early as 2012 Maes et al. demonstrated that patients with MDD are accompanied by abnormalities in the microbiota-gut-brain axis, as evidenced by elevated IgM/IgA of some gram-negative bacterial lipopolysaccharides (LPS) in serum (24). Studies have shown that many immune disorders have a high chance of co-morbidities with MDD, such as inflammatory bowel disease, rheumatoid arthritis, etc (25). The results also show that DEGs are enriched in immune response regulation signal pathway. A previous study analyzed the whole blood of MDD patients with RNA-Seq and pathway, and the results showed that the immune system-related pathways of MDD patients were up-regulated, such as Interferon alpha/beta signaling and cytokine signaling (26). Another study showed that a variety of complement pathway related proteins may regulate the immune pathogenesis of major depression (27).

CluGO analysis of the 21 shared genes showed that 70% of the genes were focused on positive cellular regulation in response to macrophage colony-stimulating factor stimulation. Macrophage colony-stimulating factor (M-CSF) has been referred to as a “secret weapon” (28) and “master regulator” (29) because of its central role in regulating various immune cells, linking innate and adaptive immunity. A study of 16 cytokines and growth factors in patients with MDD showed that M-CSF was elevated at both baseline and in response to stress (30). However, another study showed no change in M-CSF in patients with MDD (31). 20% of these genes were associated with peptide antigen assembly with MHC class II protein complex. Major histocompatibility complex (MHC) Class II molecules are primarily involved in antigen presentation. During tissue or organ transplantation, class II molecules are important target antigens that cause transplant rejection, including host-versus-graft and graft-versus-host responses. In the immune response, class II antigens are mainly responsible for coordinating the interactions between immune cells and regulating the humoral and cellular immune responses. The findings of a study showing reduced expression of MHC II-related genes in the brain in MDD also support the long-held hypothesis that altered immune function is associated with the pathophysiology of psychiatric disorders (32).

Several previous studies have also reported some characteristics of diagnostic biomarkers for MDD. For example, the authors of the original data reported that a total of 165 adaptive immunity-related genes were differentially expressed in the dataset GSE98793, and they found that the area of the higher AUC curve in the combination consisting of 165 genes was 0.71 (33). In addition, Chan et al. identified an optimal panel of 33 immunoneuroendocrine biomarkers with moderate to good performance in distinguishing depressed patients from controls (0.69 < AUC < 0.86) (34). Furthermore, Papakostas et al. reported high diagnostic performance of serum levels of nine biomarkers (alpha1 antitrypsin, apolipoprotein CIII, brain-derived neurotrophic factor, cortisol, epidermal growth factor, myeloperoxidase, prolactin, resistin and soluble tumor necrosis factor alpha receptor type II) in independent samples from patients with MDD, with sensitivity and specificity of > 80% (35). In contrast, we found in the present study that there were 4 hub genes with an AUC of 0.92 in the GSE76826 dataset. Our observations suggest that the combination of biomarkers showed good diagnostic performance.

MDD is a complex polygenic disease. In our study, RT-qPCR analysis showed that compared with HCs, the expression of IL7R in MDD patients was significantly down-regulated (*p < 0.05*), while the expression of TLR2, RETN and HP was significantly up-regulated. Of these four genes, TLR2, RETN and HP, which have been previously reported to have a clear correlation with MDD. Toll-like receptors (TLRs) play a central role in innate immunity by recognizing pathogens and damage-associated molecular patterns and are associated with a wide range of inflammatory and autoimmune disorders (36). TLR profiles predict response to antidepressant treatments. Elevated levels of TLR2 may lead to suicidality in patients with MDD (37), and depression patients experience a decrease in TLR2 levels after treatment (38). Our KEGG analysis also showed that immunity and inflammation are important pathways in MDD. Therefore, TLR2 may be a key mechanism leading to MDD and immunity. The GSEA results showed that TLR2 was associated with acute myeloid leukemia (AML), and the study demonstrated a significant association between TLR2 polymorphisms and the risk of serious infections in AML patients (39). Resistin (RETN) is a peptide secreted by adipocytes that plays a role in metabolism (40). A recent meta-analysis showed that individuals with MDD have lower serum levels of resistin compared to healthy individuals (41). RETN was found to be associated with free cortisol concentrations and treatment outcomes in patients with MDD (42). In addition, RETN is involved in the interrelationship between MDD and diabetes (43). GSEA results showed that RETN was related to the biological process of RNA degradation.

Haptoglobin (HP) is a plasma protein that binds to free hemoglobin and prevents hemoglobin-driven oxidative stress. HP plays an important role in regulating the immune response (44). An increasing number of studies have reported elevated levels of methemoglobin in patients with MDD, and methemoglobin levels are significantly correlated with the number of circulating immune cells (e.g., leukocytes, monocytes, and neutrophils) (45, 46). Notably, another variant of HP is the protein called “zonulin,” which is a tight junction protein that regulates the intestinal and blood-brain barriers (47). Zonulin has also been reported to be overexpressed in the plasma of patients with MDD, suggesting that the microbiota-gut-brain axis may underlie the pathogenesis of MDD. In our study, the results of GSEA showed that the gene was related to FC gamma r mediated phagocytosis, which may be involved in the occurrence of diseases through this pathway. IL-7, also known as lymphopoietin, belongs to the IL-2/IL-15 family of cytokines (48). IL-7 binds to IL-7R, which consists of a high-affinity α subunit (CD127) and a common γ chain (49). IL-7R is physiologically expressed on CD4 and CD8 T cells and myeloid cells, but not on human B cells (50). IL-7 is thought to support aberrant immune activity in autoimmune diseases such as diabetes mellitus and multiple sclerosis (51). In our study, IL-7R was shown to be highly correlated with immune cell T cells CD8, consistent with the above study. However, the relationship between IL7R and MDD has not been reported. IL7R was decreased in MDD patients and enrichment analyses showed that the gene was involved in cellular functions, such as being involved in the regulation of the activity of RNA polymerase transcription factors.

In addition, the analysis of hub genes’ pathways by GO and KEGG shows that it mainly focuses on PI3K-Akt signaling pathway, Toll-like receptor signaling pathway and JAK-STAT signaling pathway. Many pathways are related to immune system or signal transduction. PI3K-Akt pathway is an intracellular signal transduction pathway, which is involved in many biological processes, including metabolism, proliferation, cell survival, growth and angiogenesis (52). PI3K-Akt pathway also contributes to immune regulation, and participates in chemotaxis and phagocytosis of neutrophils, activation of B cell receptor signal transduction and maturation of dendritic cells (53). Previous studies have shown that Toll-like receptor signaling pathway induces the production of pro-inflammatory cytokines by activating NF-κB and mitogen-activated protein kinase (MAPK), which leads to the rapid activation of innate immunity. This pathway is also related to PI3K-Akt signaling pathway and JAK-STAT signaling pathway, apoptosis, complement and other processes (54). Our study identified more immune signaling pathways related to MDD, which is helpful to better study the immune mechanism of MDD development.

Over the past few decades, a growing body of evidence has emphasized the role of immune mechanisms in the development of MDD. A variety of immune cells have been implicated in the development of depression (55). Therefore, it is important to assess the relationship between MDD and immune cells. By using the CIBERSORT algorithm, our analysis identified multiple immune cell subtypes in MDD cases, further revealed significant differences in immunity between MDD and normal samples. In particular, the proportions of NK cells resting, Neutrophils, and Macrophages M0 were higher in MDD patients than in healthy controls, whereas the proportions of T cells CD8 and Mast cell resting were relatively low. Our findings are consistent with a recently published study showing that depressed patients have over-expression of innate immunity genes and under-expression of genes involved in adaptive immunity (33). We further correlated the four genes with these five immune cells and showed that TLR2 had strong correlations with NK cells resting.

Although the pharmaceutical industry has invested heavily, the success rate of new drug development continues to decline (56). Therefore, it is necessary to set goals with higher probability of success as soon as possible. Herbs with antidepressant effects, including prescription drugs, individual herbs and phytochemicals, are also widely used to treat depression. In this study, we used HIT to screen the herb components of TLR2 and IL7R, and to make molecular docking. Apicidin is a histone deacetylase inhibitor. A previous study showed that Apicidin treatment has the potential to reverse learning and memory disorders in mice with Alzheimer’s disease (57). However, there are few studies on Apicidin and MDD, and MDD and Alzheimer’s disease have many common pathogeneses. Whether we can talk about the use of Apicidin in the treatment of MDD in the future needs further study. Various studies have shown that Baicalin can improve the depression-like behavior in animal models by regulating HPA axis, promoting neurogenesis, improving mitochondrial dysfunction, inhibiting neuronal inflammation and oxidative stress, and weakening neuronal apoptosis (58). These flavonoids exist in the roots of Scutellaria baicalensis Georgi, a traditional Chinese medicine. Scutellaria baicalensis Georgi is one of the components of Xiaochaihu Decoction, which is widely used in clinical depression in China. Corilagin is a polyphenol monomer isolated from Phyllanthus urinaria, which has many pharmacological properties, including anti-tumor, anti-oxidation and anti-inflammatory effects. Previous studies have reported that Corilagin can alleviate inflammatory reaction by inhibiting TLR3 signaling pathway and reducing the release of inflammatory cytokines (59). However, the relationship between Corilagin and MDD has not been clarified, which can be used as our future research direction.

One limitation of our analysis is that it only shows that the identified drugs may be useful for the mental illness under consideration, but we are not sure which specific symptoms these drugs can solve. Further research is still needed to deepen our understanding of the safety, contraindications, interactions with other drugs and the fine molecular mechanism of potential beneficial effects of these extracts. In particular, this herbal plant may also act through unexplored biological pathways, which are related to various mental disorders.

Our study also has some limitations. Compared with previous WGCNA analyses of other diseases, our sample size was insufficient and there may be some bias. We have identified many new hub genes and enrichment pathways, however, the molecular mechanism of how it plays its role remains unclear. In this study, we merely analyzed the mRNA expression levels of hub genes in MDD patients and HCs, and did not undertake further exploration of the relationship between the clinical severity of patients and gene expression levels. This aspect requires further investigation in the future to enhance our understanding of the pathological mechanism of depression. In addition, the hub genes of MDD have been found to be associated with different immune factors, suggesting that they may also play important roles in the immune microenvironment. However, their specific roles remain to be further investigated.

## Conclusion

In conclusion, we identified for the first time four hub genes (TLR2, RETN, HP, and IL7R), which may be diagnostic biomarkers for MDD. In addition, we constructed an immune-related diagnostic model for MDD using Lasso regression analysis, which showed good diagnostic performance in the dataset GSE98793. Thus, our genetic characterization can provide an accurate and reliable prediction method for patients. Finally, the immune cell infiltration of MDD patients was analyzed using CIBERSORT, and correlation analysis showed that hub genes are involved in the immune response in MDD. Our study combined with further validation of blood samples from clinical patients provides strong evidence for studies exploring immune involvement in the pathophysiologic pathogenesis of MDD, which will be a new starting point. In the future, to validate the results of our analysis, it is essential to expand the sample size and conduct multi-center, large-sample randomized controlled trials alongside biological investigations. These efforts will help elucidate how hub genes and hub pathways influence the development of affective disorders. We recommend identifying subgroups of mental diseases that exhibit different phenotypes yet share common pathogenic mechanisms. This approach aims to facilitate the development of new drugs targeting shared molecular biological pathways. Furthermore, drug development should prioritize targeting these biological pathways rather than focusing solely on isolated genes or proteins.

## Data Availability

The original contributions presented in the study are included in the article/**Supplementary Material**. Further inquiries can be directed to the corresponding author.
