# Supplementary material for: Immune-related gene characterization and biological mechanisms in major depressive disorder revealed based on transcriptomics and network pharmacology
Source: Front Psychiatry. 2024 Dec 6;15:1485957. doi: 10.3389/fpsyt.2024.1485957 (PMC11659238; doi:10.3389/fpsyt.2024.1485957)
Supplement: Supplementary file 1 [file Supplementaryfile1.zip › Supplementary Figure 1.DOCX]

**Figure S1** 14 herbal compound structures related to TLR2 (A-N) and 1 herbal compound structure related to IL7R (O).


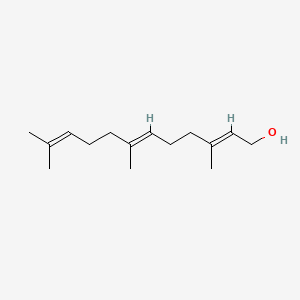


(A) The structure of Farnesol (C0072).


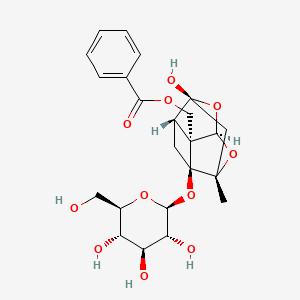


(B) The structure of Paeoniflorin (C0282).


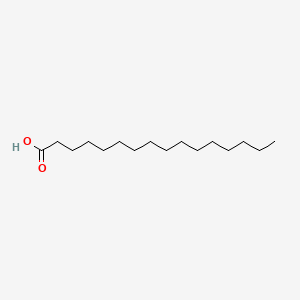


(C) The structure of Palmitic Acid (C0285).


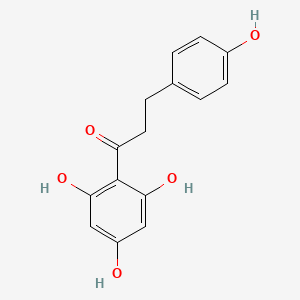


(D) The structure of Phloretin (C0302).


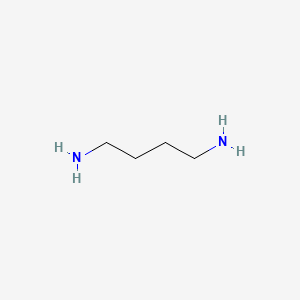


(E) The structure of Putrescine (C0340).


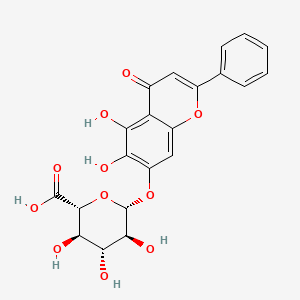


(F) The structure of Baicalin (C0721).


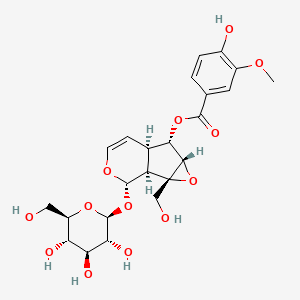


(G) The structure of Picroside Ii (C0828).


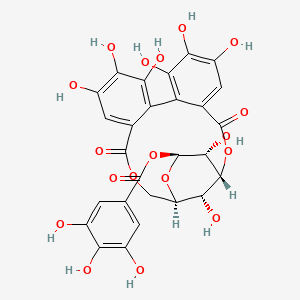


(H) The structure of Corilagin (C0832).


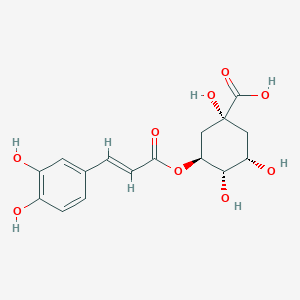


(I) The structure of Chlorogenic Acid (C0833).


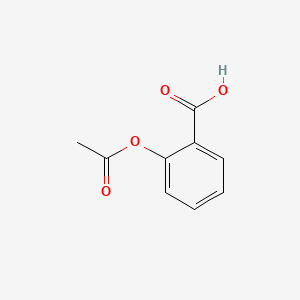


(J) The structure of Aspirin (C0934).


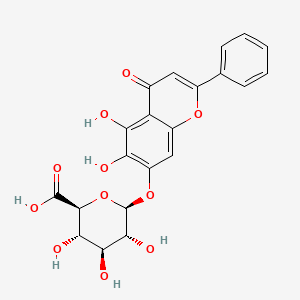


(K) The structure of Baicalin (C1125).


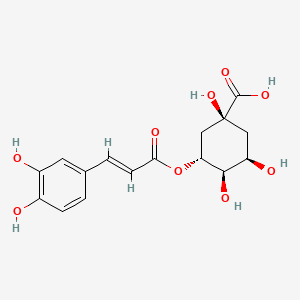


(L) The structure of Chlorogenic Acid (C1231).


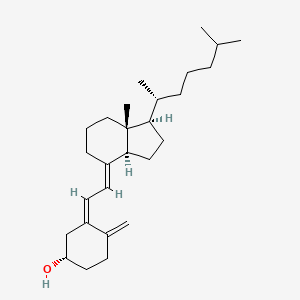


(M) The structure of Cholecalciferol (C1233).


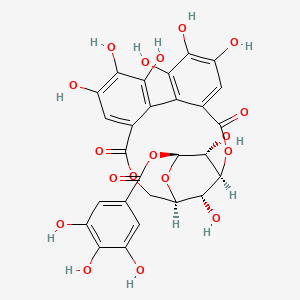


(N) The structure of Corilagin (C1252).


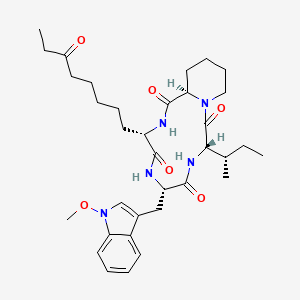


(O) The structure of Apicidin (C0803).
