# Supplementary material for: Immune-related gene characterization and biological mechanisms in major depressive disorder revealed based on transcriptomics and network pharmacology
Source: Front Psychiatry. 2024 Dec 6;15:1485957. doi: 10.3389/fpsyt.2024.1485957 (PMC11659238; doi:10.3389/fpsyt.2024.1485957)
Supplement: Supplementary file 1 [file Supplementaryfile1.zip › Supplementary Figure 2.DOCX]

**Figure S2** Molecular docking of TLR2 with 14 herbal compounds (A-N) and IL7R with 1 herbal compound (O).


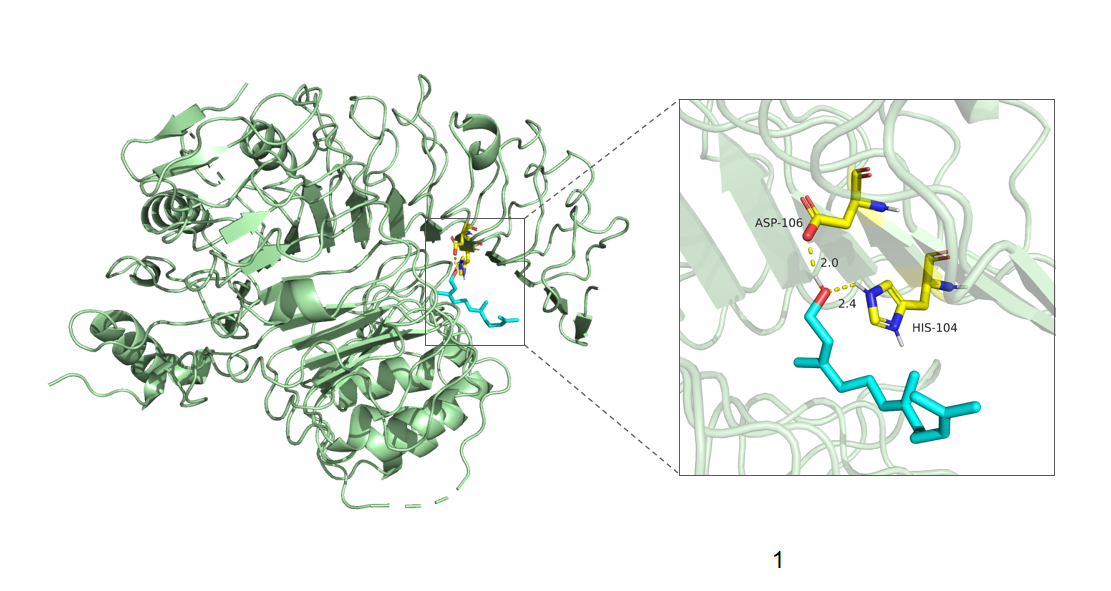
(A) Molecular docking pattern of TLR2 with Farnesol (C0072).


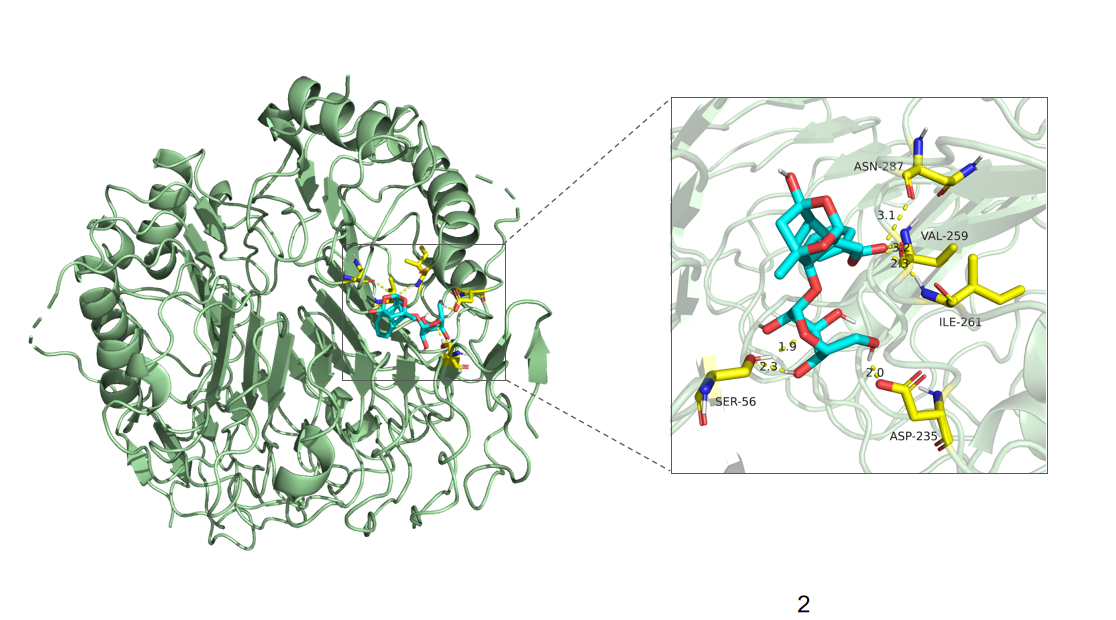


1. Molecular docking pattern of TLR2 with Paeoniflorin (C0282).


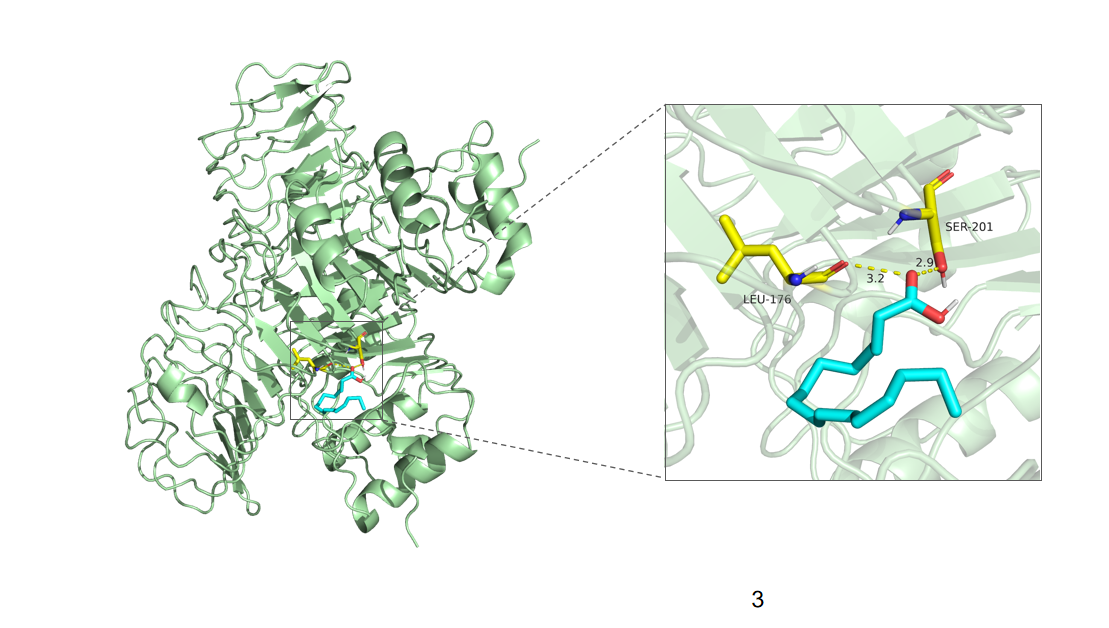


1. Molecular docking pattern of TLR2 with Palmitic Acid (C0285).


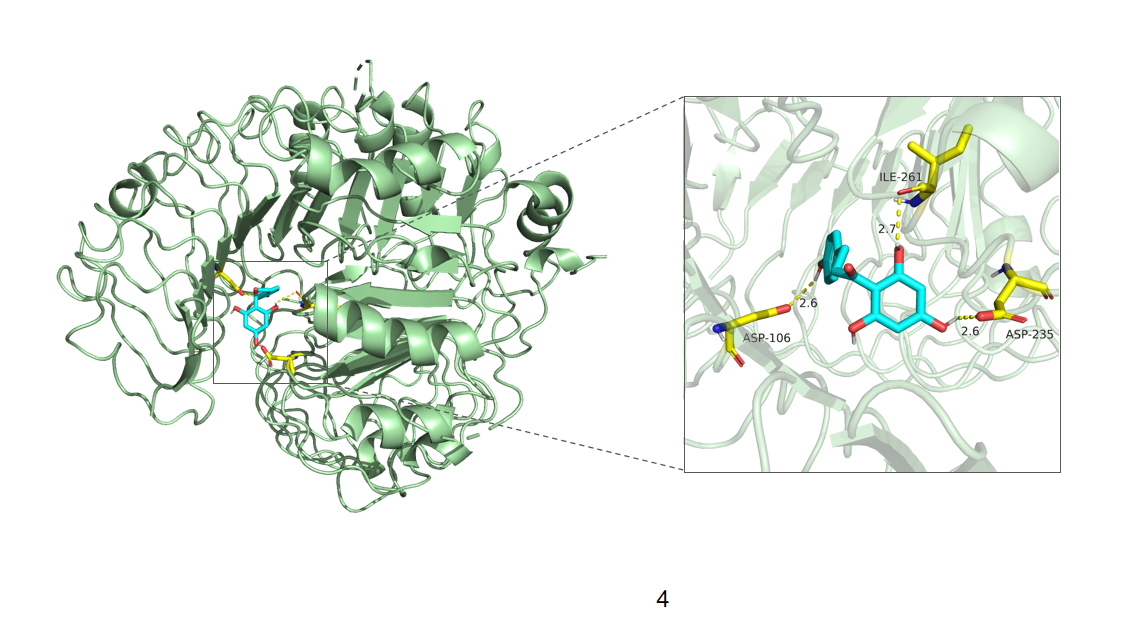


1. Molecular docking pattern of TLR2 with Phloretin (C0302).


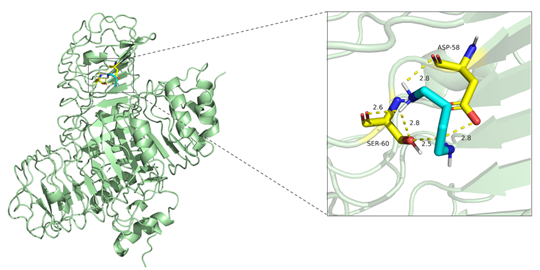


1. Molecular docking pattern of TLR2 with Putrescine (C0340).


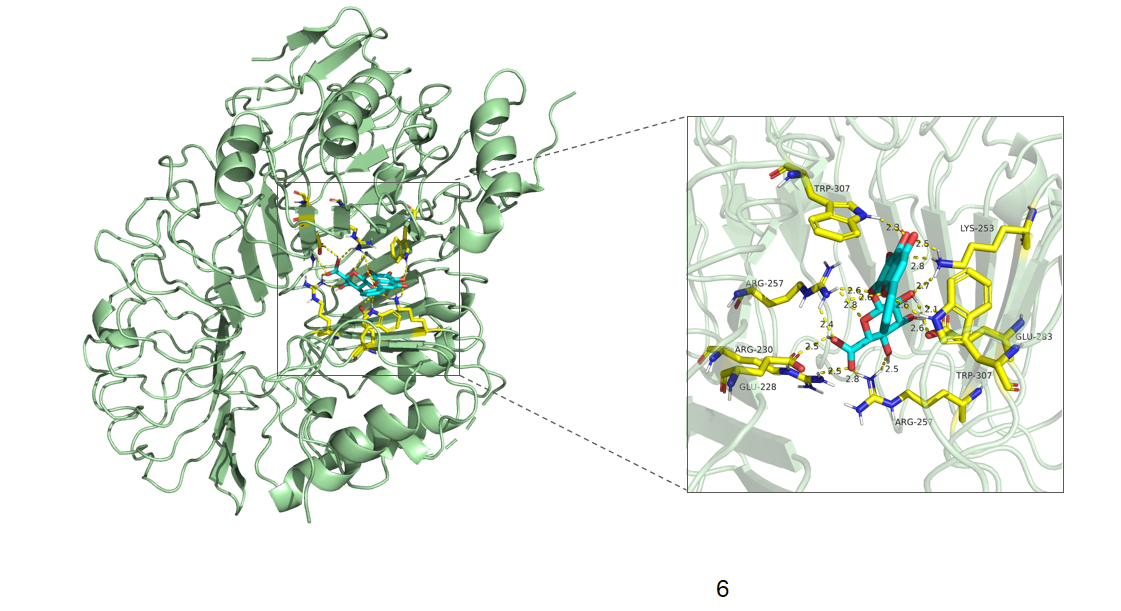


1. Molecular docking pattern of TLR2 with Baicalin (C0721).


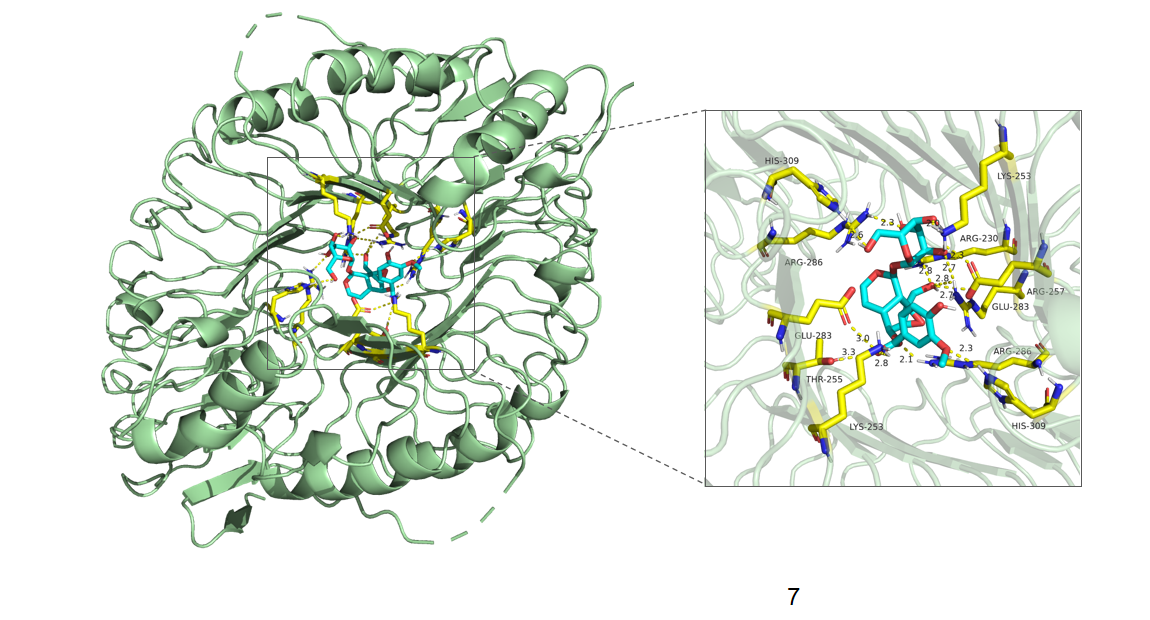


1. Molecular docking pattern of TLR2 with Picroside Ii (C0828).


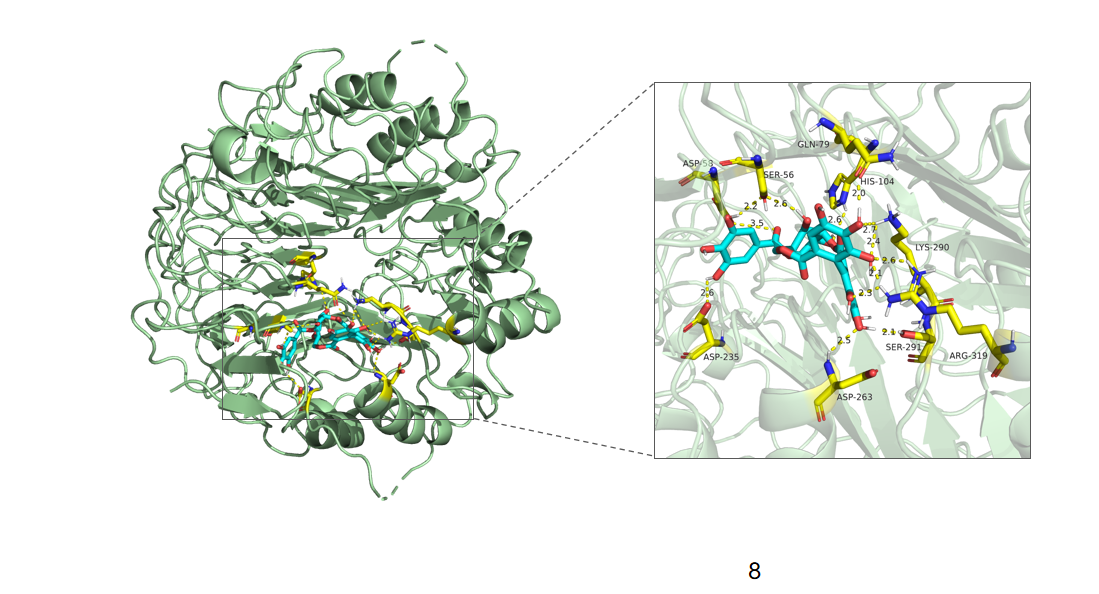


1. Molecular docking pattern of TLR2 with Corilagin (C0832).


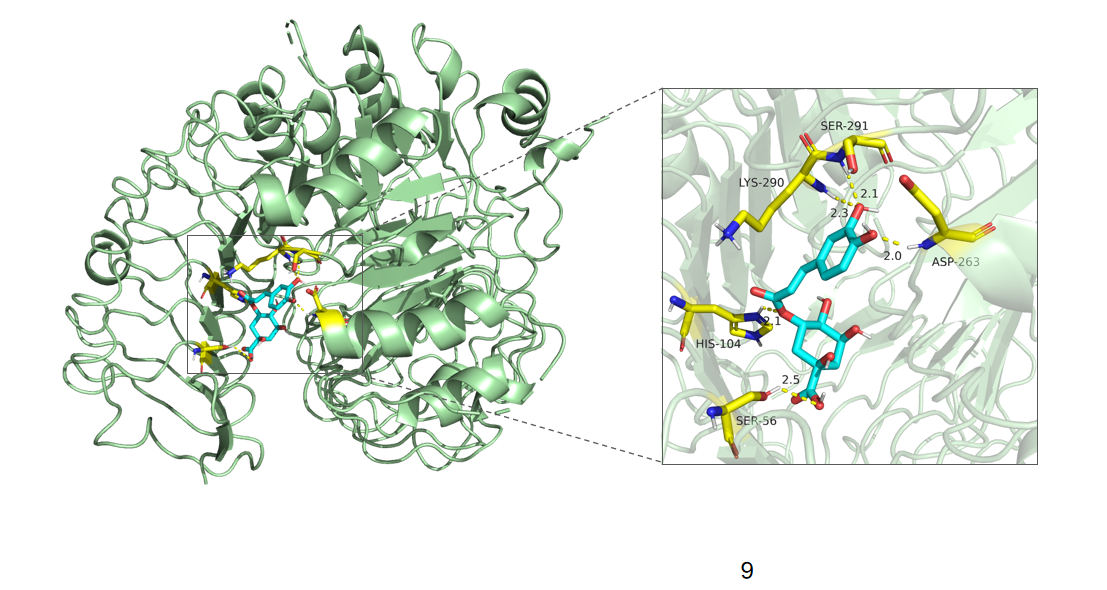


1. Molecular docking pattern of TLR2 with Chlorogenic Acid (C0833).


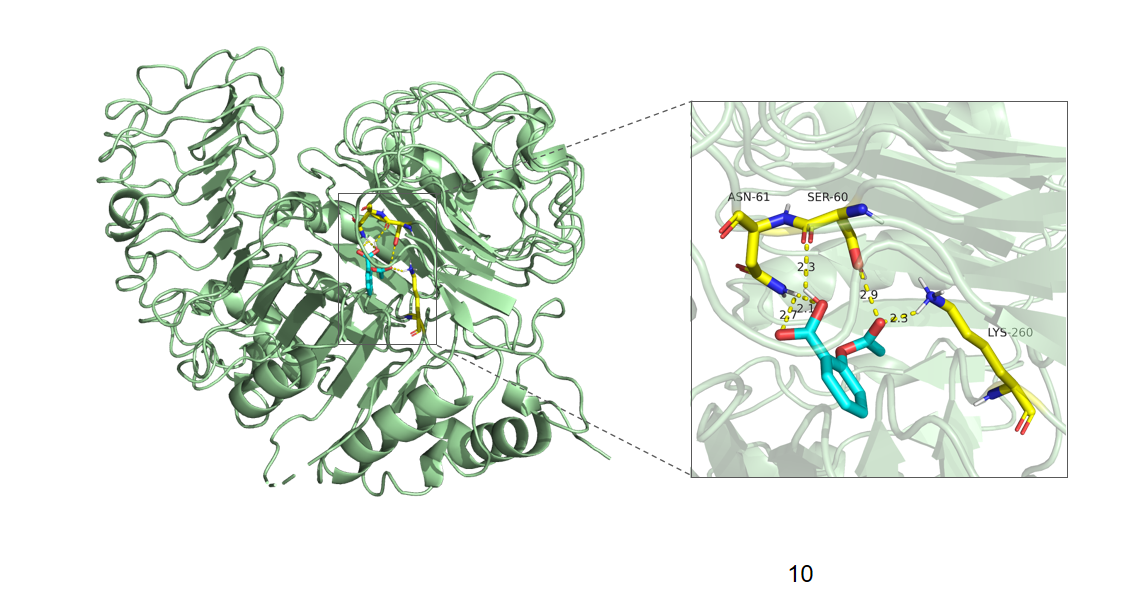


1. Molecular docking pattern of TLR2 with Aspirin (C0934).


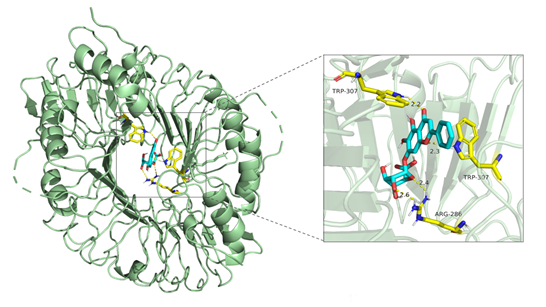


1. Molecular docking pattern of TLR2 with Baicalin (C1125).


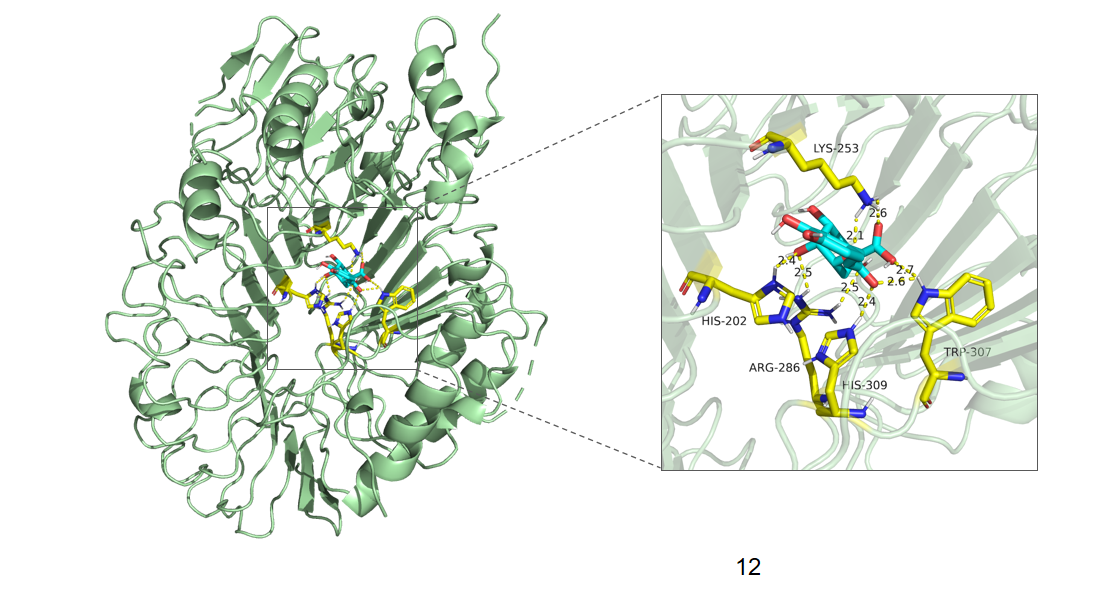
(L) Molecular docking pattern of TLR2 with Chlorogenic Acid (C1231).


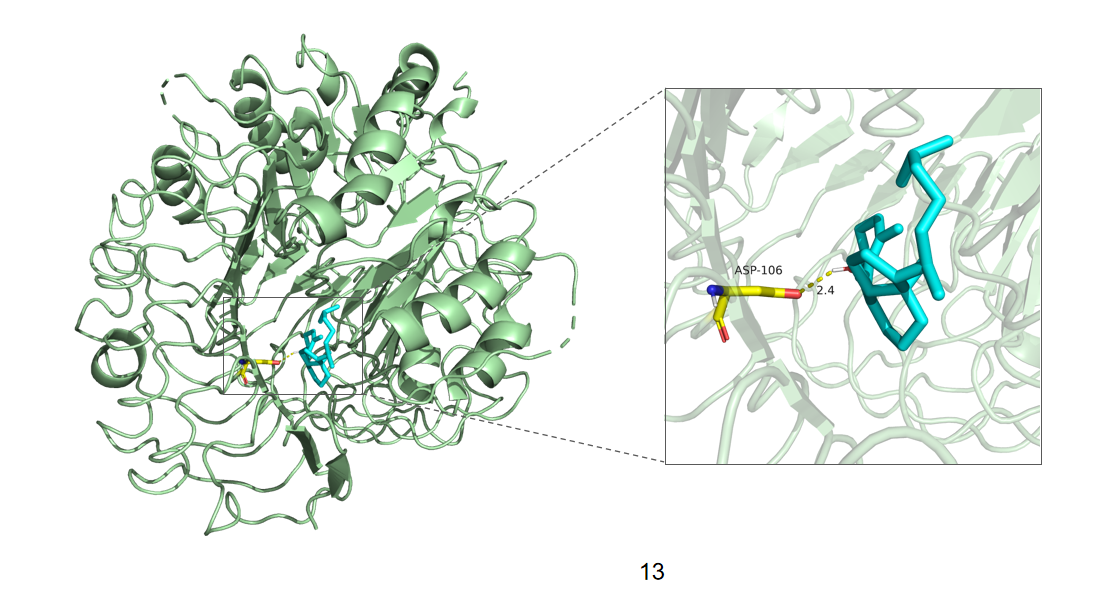


(M) Molecular docking pattern of TLR2 with Cholecalciferol (C1233).


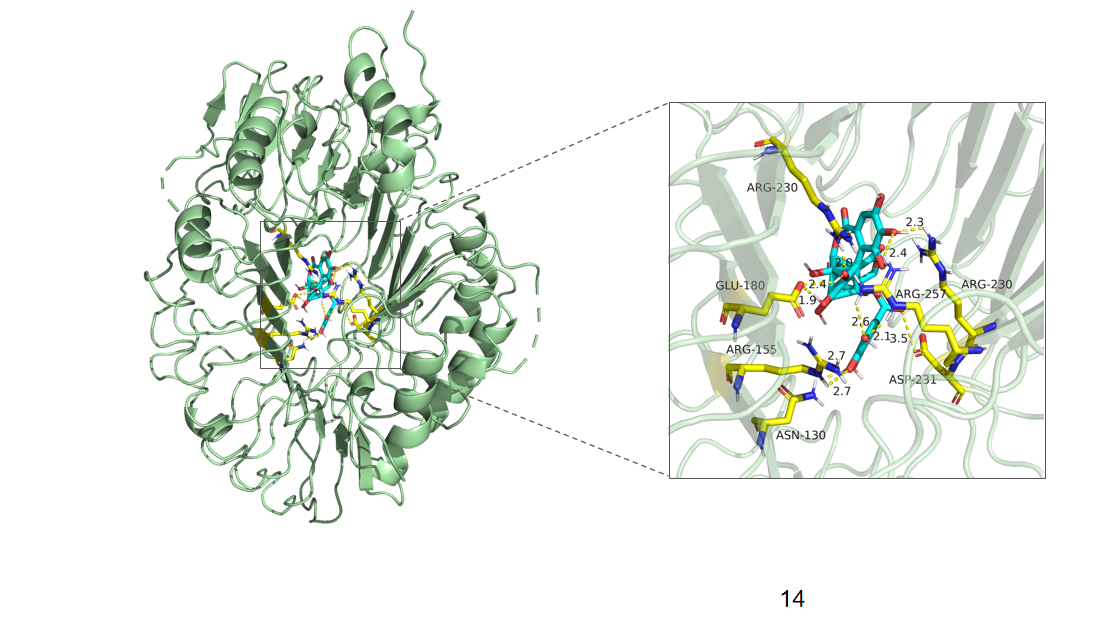


(N) Molecular docking pattern of TLR2 with Corilagin (C1252).


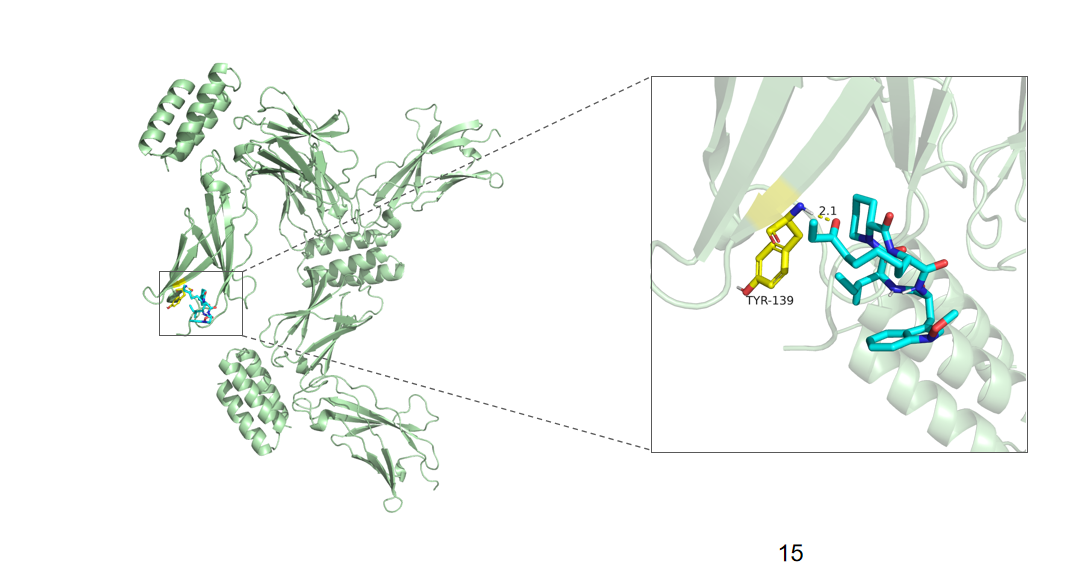
(O) Molecular docking pattern of IL7R with Apicidin.
