# Supplementary material for: Immune-related gene characterization and biological mechanisms in major depressive disorder revealed based on transcriptomics and network pharmacology
Source: Front Psychiatry. 2024 Dec 6;15:1485957. doi: 10.3389/fpsyt.2024.1485957 (PMC11659238; doi:10.3389/fpsyt.2024.1485957)
Supplement: Supplementary file 1 [file Supplementaryfile1.zip › Supplementary Table 7.DOCX]

**Table S7** Descrption of the compounds corresponding to the two hub genes.

| **Target** | **ID** | **Pubchem ID** | **Common name** | **Formula** | **Binding energy (kcal/mol)** |
| --- | --- | --- | --- | --- | --- |
|  |  |  |  |  |  |
| TLR2 | C0072 | CID:445070 | Farnesol | C_15_H_26_O | -6.0 |
| TLR2 | C0282 | CID:46882877 | Paeoniflorin | C_23_H_28_O_11_ | -8.8 |
| TLR2 | C0285 | CID:985 | Palmitic Acid | C_16_H_32_O_2_ | -4.9 |
| TLR2 | C0302 | CID:4788 | Phloretin | C15H14O5 | -7.4 |
| TLR2 | C0340 | CID:1045 | Putrescine | C_4_H_12_N_2_ | -3.5 |
| TLR2 | C0721 | CID:1549028 | Baicalin | C_21_H_18_O_11_ | -9.8 |
| TLR2 | C0828 | CID:11944602 | Picroside Ii | C_23_H_28_O_13_ | -8.9 |
| TLR2 | C0832 | CID:12303949 | Corilagin | C_27_H_22_O_18_ | -11.8 |
| TLR2 | C0833 | CID:12310830 | Chlorogenic Acid | C_16_H_18_O_9_ | -7.9 |
| TLR2 | C0934 | CID:2244 | Aspirin | C_9_H_8_O_4_ | -5.8 |
| TLR2 | C1125 | CID:64982 | Baicalin | C_21_H_18_O_11_ | -9.3 |
| TLR2 | C1231 | CID:1794427 | Chlorogenic Acid | C16H18O9 | -8.0 |
| TLR2 | C1233 | CID:5280795 | Cholecalciferol | C27H44O | -8.0 |
| TLR2 | C1252 | CID:73568 | Corilagin | C27H22O18 | -10.2 |
| IL7R | C0803 | CID:6918328 | Apicidin | C_34_H_49_N_5_O_6_ | -3.4 |
